# Curative Potential of Substances with Bioactive Properties to Alleviate Cd Toxicity: A Review

**DOI:** 10.3390/ijerph191912380

**Published:** 2022-09-28

**Authors:** Miroslava Požgajová, Alica Navrátilová, Marek Kovár

**Affiliations:** 1 AgroBioTech Research Centre, Slovak University of Agriculture in Nitra, Tr. A. Hlinku 2, 94976 Nitra, Slovakia; 2 Institute of Nutrition and Genomics, Faculty of Agrobiology and Food Resources, Slovak University of Agriculture in Nitra, Tr. A. Hlinku 2, 94976 Nitra, Slovakia; 3 Institute of Plant and Environmental Science, Faculty of Agrobiology and Food Resources, Slovak University of Agriculture in Nitra, Tr. A. Hlinku 2, 94976 Nitra, Slovakia

**Keywords:** cadmium, toxicity, oxidative stress, bioactive substances

## Abstract

Rapid urbanization and industrialization have led to alarming cadmium (Cd) pollution. Cd is a toxic heavy metal without any known physiological function in the organism, leading to severe health threat to the population. Cd has a long half-life (10–30 years) and thus it represents serious concern as it to a great extent accumulates in organs or organelles where it often causes irreversible damage. Moreover, Cd contamination might further lead to certain carcinogenic and non-carcinogenic health risks. Therefore, its negative effect on population health has to be minimalized. As Cd is able to enter the body through the air, water, soil, and food chain one possible way to defend and eliminate Cd toxicities is via dietary supplements that aim to eliminate the adverse effects of Cd to the organism. Naturally occurring bioactive compounds in food or medicinal plants with beneficial, mostly antioxidant, anti-inflammatory, anti-aging, or anti-tumorigenesis impact on the organism, have been described to mitigate the negative effect of various contaminants and pollutants, including Cd. This study summarizes the curative effect of recently studied bioactive substances and mineral elements capable to alleviate the negative impact of Cd on various model systems, supposing that not only the Cd-derived health threat can be reduced, but also prevention and control of Cd toxicity and elimination of Cd contamination can be achieved in the future.

## 1. Introduction

Cadmium (Cd) is a non-essential metal with an unknown physiological function in the organism. It represents a great concern as it was shown to cause a number of adverse health effects in humans. Cadmium has acquired great attention, after reports in the 1960s, to be responsible for a painful bone disease seriously expanded in highly polluted areas in Japan. In this area, consumption of the polluted food and water for more than three decades led to considerable Cd accumulation in the body leading to the development of an osteoporosis-like disease “itai–itai” that can be descriptively translated as “it hurts–it hurts disease” [1,2]. Although Cd is a naturally occurring heavy metal, over 90% of Cd in the surface environment derives from anthropogenic activities. The major sources of environmental contamination by cadmium include fossil-fuel combustion, cement manufacture, phosphate fertilizer production, metallurgical work, sewage sludge and waste deposition [3]. As Cd is characteristic for its slow degradability it persists in the environment for very long time, which increases the possibility of its absorption by plants and other organisms and its accumulation in living systems. Moreover, due to the high soil-to-plant transfer of Cd, its access by humans is almost unavoidable. The food chain, together with cigarette smoke and polluted air, are the primary sources of Cd intake by humans, leading to the increase in Cd levels in blood, urine, and tissues. Cadmium-derived food contamination is a complex issue related to geographical localization, Cd bioavailability from the soil, soil composition, variety of cultivated crops, plant genetics, local agricultural practices, postharvest manipulation with raw material, and food preparation [4].

The purpose of this work was to perform a detailed and broad literature search dedicated to mitigation strategies of Cd toxicity through food-derived compounds with bioactive properties. The toxicity of Cd was described in variable model systems ranging from plants, and microorganisms, to animals. Elimination of Cd toxicity and understanding of the way it affects living systems may contribute to the development of strategies how to protect the population from Cd toxicity. Therefore, this work includes a broad range of studies considering the impact of Cd and the possibilities of its alleviation in most types of biological samples. An extensive literature search originating from journals indexed in international databases such as Medline/PubMed, Web of Science, Science Direct/Elsevier, and Springer was employed. Additionally, Google books and Google Scholar were also taken into consideration. The eligible literature sources cited in the review are publications solely written in the English language and dated from 2003 to 2022; however, the vast majority are dated within the last five years. The following combinations of terms (keywords) were applied for the literature search: “Cadmium”, “toxicity”, “itai-itai”, “oxidative stress”, “free radical”, “gastrointestinal”, “renal toxicity”, “hepatotoxicity”, “cytotoxicity”, “environmental contamination”, “mitigation”, “mineral elements”, “macroelements”, “microelements”, “vitamins”, “bioactive compounds”, “antioxidant”, “plants”, “animals”, and “microorganisms”. Unpublished works or personal communications, case reports or letters, and articles without available full-length were excluded. Taken together, a total of 200 relevant literature sources were collected and processed for the review.

## 2. Cadmium Toxicity

The severity of the Cd impact on human health closely relates with its extra-long half-life period of about 20–30 years in humans, in combination with the low Cd excretion rate from tissues and organs that leads to accumulation of Cd in the body resulting in its long-term exposure causing variable adverse effect on health [5]. To minimize negative effects of Cd to human population, critical amounts of Cd in foodstuffs and the environment have been established [6,7]. Provisional tolerable monthly intake was established according to epidemiological studies by Food and Agriculture Organization of the United Nations/World Health Organization (FAO/WHO) expert committee and was set up to 25 μg/kg body weight. It represents daily intake of 0.83 μg/kg body weight/day or 58 μg/day for a 70 kg person. A urinary Cd threshold level is 5.24 μg/g creatinine. Moreover, the safety limit for Cd in the air was set up to 5.0 ng/m^3^ (annual average), in drinking water 3 μg/L and 3 mg/kg is a limit for soils that are actively used for crop production [7,8].

Transportation of Cd usually bound to a sulfhydryl group in the body directs predominantly to the soft tissues such as liver (30%) and kidney (30%) and the remaining Cd is distributed throughout the body [9]. Although the primary organ attacked by Cd is the kidney, Cd has been shown to alter the structure and function of the liver, lung, pancreas, reproductive organs, placenta, bones, and the hematopoietic, nervous, and cardiovascular systems. Moreover, Cd-mediated oxidative stress causes impairment of oocyte maturation, decrease in fertility or sexual dysfunction, and impairs embryonic development that often leads to prenatal death [10,11,12,13]. Cadmium also represents a direct toxicant for rat Leydig cells as it enhances formation of DNA single strand breaks which in turn decreases percentage of normal cells that led to the decrease in testosterone secretion [14]. In addition, even a non-toxic dose of Cd has been shown to be an endocrine-disrupting element as it changes the gene expression pattern in the testes which results in increased disposition to testicular autoimmunity. Moreover, Cd can directly bind to estrogen and androgen receptors, which in turn potentiates estrogen- and androgen-like activities [15,16].

**Figure 1:**
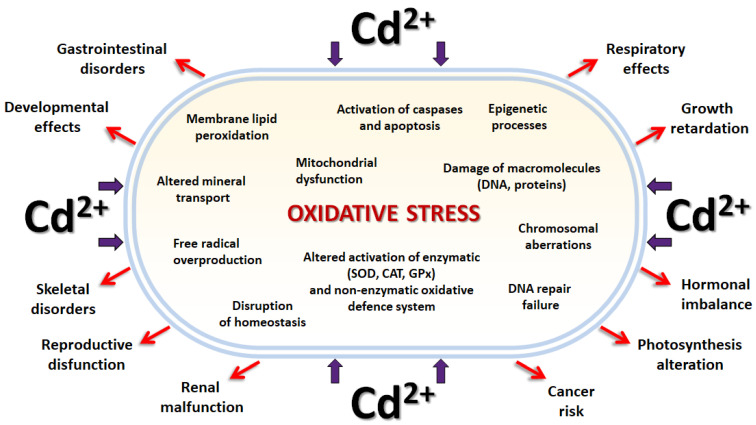
Cadmium toxicity. Schematic illustration of the hazardous effects of Cd toxicity on living organisms. Cd overexposure of the organism leads to severe cellular alterations predominantly due to increased production of free radicals and impaired antioxidant defense system resulting in oxidative stress consequently leading to membrane lipid peroxidation, damage of macromolecules, chromosomal aberrations, DNA repair failure, and metabolic deteriorations disrupting cellular homeostasis, activation of caspases, and apoptosis. Defects in cell function cause injury to the organism that may result in gastrointestinal disorders, developmental defects, skeletal alterations, reproductive dysfunction, renal malfunction, cancer risk, respiratory effects, growth retardation, hormonal imbalance, or altered photosynthesis.

As cadmium is one of the most toxic elements, it is in the center of scientific interest already for several decades. These days, the Cd-mediated DNA alterations can be considered as the prominent and concerning effects of the metal. Exposure to Cd has been associated with direct cytotoxic effects resulting in carcinogenic, apoptotic, and/or necrotic events (Figure 1). Cd has been shown to induce DNA damage in various model systems and through variable mechanisms. Significant genetic alterations in Cd-exposed seedlings of *Arabidopsis thaliana* plantlet shoots were detected with the use of a random amplified polymorphic DNA (RAPD) test [17]. Moreover, cadmium treatment of C57BL/6 pregnant mice causes DNA damage in the embryonic cells and triggers activation of the apoptotic pathway as it shifts the *Bcl-2/Bax* equilibrium through upregulation of *p53*, *p21*, and *Bax* transcription levels and downregulation of *Bcl-2* [18]. In addition, Cd-mediated DNA fragmentation via induction of oxidative stress resulting in the cell apoptotic events have been reported in the hepatopancreas of the shellfish *Meretrix meretrix* [19], in the testes of frog *Rana limnocharis* [20], in osteoblast cell lines [21,22], but also in human liver carcinoma cells [23]. The present knowledge about the mechanism of Cd-mediated mutagenicity has been extensively reviewed [24,25]. The carcinogenic activity of Cd has been substantiated by its ability to inhibit the post-replication mismatch repair of DNA [26,27] and the ability to prevent the cell cycle arrest [28,29] together resulting in a high level of genetic instability. In the majority of the published scientific papers, Cd-induced DNA damage tightly correlate with Cd-mediated oxidative stress [30,31,32,33]. Although Cd does not enhance the production of reactive oxygen species (ROS) directly, it interferes with the antioxidant system of the cell which results in the accumulation of ROS such as the hydroxyl radical, superoxide anion, or hydrogen peroxide which results in oxidative DNA damage. Other Cd-derived cell impairments are related to the cellular ionome balance instability affecting cell homeostasis [34,35,36,37,38], and to the Cd-mediated epigenetic modifications that change the expression of genes [37,39,40,41,42].

## 3. Mitigation of Cd Toxicity via Food Supplementation

According to studies from authors around the globe, the permissible concentration limits of Cd ingestion from food or water have often been exceeded [43,44,45]. Moreover, Cd is not involved in any physiological process and is known only for its toxic impact on living organisms; thus, its negative effect on population health has to be minimalized. In the kinetics and metabolism of Cd sequestration in the body, play metallothioneins (MT) a critical role as they bind Cd to form CdMT and thereby prevent the immediate toxic effect of Cd, reviewed by [46]. However, higher concentration or long-term exposure of Cd exceeds the MT capacity; therefore, mitigation strategies to eliminate the impact of Cd on the organism need to be applied. One of the most promising strategies represents the food supply system. Various foods including Tualang honey [47], fruits [48], and olive or colocynth oil [49] have been shown to trigger beneficial features against Cd-mediated toxicity. However, not the food itself but the biologically active substances it contains, in particular, the antioxidative nutrients are responsible for the elimination of Cd toxicity.

### 3.1. Vitamins

#### 3.1.1. Vitamin C (Ascorbic Acid)

Vitamin C (L-ascorbic acid, ascorbate, AsA) is a prominent antioxidant that plays an unreplaceable role in the sequestration of excessive ROS in cells exposed to abiotic stress. Research studies that involve genetic manipulations of genes responsible for biosynthesis, catabolism, and recycling of AsA to modify AsA synthesis in plants revealed that AsA is able to counteract or prevent various stresses including those caused by metals such as Cd [50]. Furthermore, it was necessary to investigate if also exogenous application of AsA on plant model systems has the capacity to protect them from Cd toxicity. Indeed, foliar application of AsA (0.1, 0.3, or 0.5 mM) on maize (*Zea mays* L.) regenerated growth and proliferation, photosynthetic capacity, and protein concentration in maize plants exposed to Cd (50 mg/kg soil of CdSO_4_ for 4 weeks). Cd exposure induced oxidative stress to maize plants detected by enhanced malondialdehyde (MDA) contents, H_2_O_2_ accumulation, and enhanced activities of superoxide dismutase (SOD), peroxidase (POX), catalase (CAT), and glutathione reductase (GR), which were however significantly improved by AsA. Moreover, Cd uptake by maize grains was reduced upon foliar AsA application [51]. Similarly, the protective effect of AsA (250 mg/kg or 500 mg/kg through single foliar application, or seedlings preincubation with 1 mM AsA, respectively), via the AsA–GSH–NADPH cycle homeostasis maintenance to reduce ROS content was detected also upon its application on Cd-stressed oilseed rape seedlings exposed to 10 μM CdCl_2_ (*Brassica napus* L. cv. Tammi) [52] and wheat seedlings exposed to 100 μM CdCl_2_ [53].

To demonstrate the Cd toxicity on the entire homeostasis of the organism, its impact and the effect of AsA treatment were investigated by [54] on the single-celled non-pathogenic yeast *Schizosaccharomyces pombe*. Concomitant with findings performed on plant model systems, cells significantly profited from AsA treatment (10 mM AsA pretreatment for 30 min). Cells exposed to Cd (10, 20, 40, 100, or 400 μM CdCl_2_) suffered from impaired ionome balance and oxidative stress causing membrane lipid peroxidation leading to cell shape alterations which in turn caused growth retardation. AsA pre-treatment markedly improved the cell condition affected by Cd.

In addition, with the use of animal model systems Cd has been shown to cause hepatotoxicity and cardiac toxicity in rabbits. Orally administered CdCl_2_ in a dose of 1.5 mg/kg body weight (b.w.) for 28 days caused a substantial accumulation of Cd ions in the liver and heart and altered normal biochemical parameters of the liver and the blood plasma, which was, however, largely reversed by AsA (150 mg/kg b.w.) supplementation [55,56]. In the mouse model system, it was shown that CdCl_2_ exposure (100 mg/L drinking water for 8 weeks) causes vascular dysfunction closely associated with the altered antioxidant capacity of the organism. The study for the first time experimentally proves the pharmacological effects of AsA (50 or 100 mg/kg b.w.) on mitigation of oxidative damage and improvement of the vascular function of Cd-induced hypertension and vascular dysfunction in mice [57]. According to these studies, the use of AsA supplementation can be suggested as the therapeutic and prophylactic approach against Cd-induced perturbations.

#### 3.1.2. Vitamin E

The term vitamin E summarizes tocopherols and tocotrienols of which only the α-tocopherol represents vitamin E that meets the requirements for humans. It is a fat-soluble antioxidant present in all cells, responsible for the primary cell protection against membrane lipid peroxidation. In addition to its strong antioxidant feature through regulation of ROS production, vitamin E possesses immune boosting properties and modulates signal transduction and gene expression to protect the organism against cancer, and cardiovascular or neurodegenerative diseases [58,59,60]. Although vitamin E deficiency is rare, supplementation of vitamin E has been shown to substantially enhance not only the immune functions of the organism but also to improve the conditions of the organism exposed to abiotic stress. The effect of vitamin E supplementation on Cd-induced stress has been documented by a variety of aquatic and terrestrial model systems.

As aquatic animals are more likely to accumulate Cd as compared to terrestrial animals, elimination of Cd impact of fish in fish farms is necessary by any adequate method. Cd has been shown to reduce weight gain and protein concentration, while serum creatinine, AST (aspartate aminotransferase) and ALT (blood alanine aminotransferase) values increased, demonstrating alteration of the liver and kidney function in Nile tilapia fish. The authors of the study used natural clay (3% of the diet), vitamin C (100 mg/kg diet) and vitamin E (50 mg/kg diet) supplementation of the Cd (25 or 50 mg CdCl_2_/kg diet) contaminated diet. All supplemented additives enhanced growth rate, improved Cd-affected levels of serum creatinine, ALT, and AST values, and reduced residual Cd in the fish bodies. Hence, vitamin E but also vitamin C and natural clay have the ability to ameliorate the function of kidney and liver in fish exposed to Cd-induced stress [61]. Similarly, grass carp *Ctenopharyngodon idellus* exposure to Cd (intraperitoneal (i.p.) injection of 20 μM/kg CdCl_2_ and samples were collected on days 4, 8, 12, and 16 after injection) resulted in the liver, blood, and kidney toxicity. The liver toxicity has been manifested through the Cd content increase in hepatocytes, elevation of MDA concentration, increased percentage of hepatocyte apoptosis, and enhanced apoptosis-related gene mRNA transcript expression. Cd addition affected the function and morphology of the liver and altered the antioxidant defense mechanism as the activity of CAT, SOD, and GPx (glutathione peroxidase) markedly decreased. Cd content in the blood and trunk kidney also increased upon grass carp exposure to Cd, which was associated with organ damage and immunotoxicity. Vitamin E (20 IU/kg supplemented on day 4 after CdCl_2_ injection) supplementation of Cd-treated fish significantly improved the health conditions of intoxicated fish, as it led to the decrease in Cd content in the blood and tissue, stabilization of the damaged organs, and promotion of the protective antioxidant system [62,63].

Tissue toxicity and damage due to Cd-mediated stress were also detected in the terrestrial animal model systems. Hepatic toxicity of rats and mice exposed to Cd (CdCl_2_ in a dose of 5 mg/kg b.w. for 28 days or 30 ppm in drinking water for 7 weeks, respectively), has been demonstrated by the reduction in liver weight, liver histological alteration, and oxidative stress, increased Cd content in the liver, elevated ALT and AST concentrations in the blood serum, severe hepatocellular necrosis, a rupture in hepatocytes, and disarrangement of hepatic strands. Vitamin E administration (100 mg/kg b.w. or 50 IU/kg, respectively), improved Cd-derived biochemical and histopathological changes through inhibition of oxidative stress as it promotes the expression of genes encoding proteins of the Nrf2 (nuclear factor erythroid 2–related factor 2) pathway a prominent regulatory pathway that controls cellular responses to oxidants [64,65].

Renal toxicity of rats exposed to CdCl_2_ was demonstrated by several independent scientific laboratories through histological and biochemical analyses. Administration of CdCl_2_ to experimental animals was performed either through daily gavage (20 mg/kg b.w. for 30 days, 5 mg/kg b.w. for 28 days, respectively), or intraperitoneally (2 mg/kg b.w. per day for 8 days), or in drinking water (50 ppm Cd in distilled water for 20 weeks). Alterations of the activities of the enzymatic defense system including SOD, CAT, glutathione reductase (GR), GPx, glutathione-S-transferase (GST), and lipid peroxidase (LPx) upon Cd addition were determined in the kidney tissue and renal mitochondrial fractions. Additionally, Cd treatment led to its accumulation in the kidney, caused a decrease in the body and kidney weight, elevated levels of BUN (blood urea nitrogen) and SCr (serum creatinine) in the serum, and led to renal histological alterations. Cd poisoning decreased the glomerular filtration rate (GFR), but enhanced angiotensin-converting enzyme (ACE) activity, and increased blood pressure and heart rate. Vitamin E supplementation (20 mg/kg b.w., 100 mg/kg b.w., 250 mg/kg b.w., or 40 and 400 mg/kg b.w., respectively), in rats exposed to Cd, helped to protect the kidney from functional damage and to normalize renal dysfunction, inhibited apoptosis of renal cells, protected kidney from oxidative stress via enhancement of the antioxidant defense system [66,67,68,69].

Furthermore, analyses of testes of the rats exposed to Cd (either by every day injection of 2 mg/kg b.w. Cd(NO_3_)_2_ for 28 days, or by single injection of 2 mg/kg b.w. CdCl_2_ on day 30 of the experimental period, respectively), displayed apoptotic events through the mitochondrial pathway as it increased the ratio of Bay/Bcl-2, decrease in the proton pump (Na^+^/K^+^-ATPase, Ca^2+^-ATPase, and Mg^2+^-ATPase) activity resulting in the drop of sperm count, viability and motility, and the decrease in hormonal levels. Vitamin E treatment (100 UI/kg b.w. for 28 days, or 100 mg/kg b.w. for 30 days, respectively), ameliorated the toxic effect of Cd on proton pump activity in the testes, protected testes from Cd-induced apoptosis, improved testicular integrity, structure, and functions [70,71].

In general, intoxication with Cd represents a great challenge to the protective antioxidant system of the organism. Application of Cd (single intraperitoneal (i.p.) injection of 0.4 mg/kg b.w. CdCl_2_, or 1 mg/kg b.w. per day by subcutaneous (s.c.) injection of CdCl_2_ for 30 days, respectively), mediated increased production of ROS causes oxidative stress to the cell which alters the hematological and biochemical parameters, increases plasma creatinine, MDA, ALT, AST, ALP, and BUN levels, decreases GPx, CAT, GP, GST and SOD activity. Vitamin E (single i.p. injection of 20 UI/kg b.w. or 60 mg/kg b.w. by s.c. injection for 30 days, respectively), treatment exhibits a protective role on the toxic effects of Cd on the hematological values, and also on the components of the antioxidant defense system [72,73,74].

The toxic effect of Cd on the tested systems and its alleviation by vitamin supplementation is summarized in Table 1.

### 3.2. Mineral Elements

#### 3.2.1. Selenium

An essential trace element, selenium, can be naturally found in soil, water, and some food. For the normal function of metabolism, an everyday intake of a small amount of Se is required. The adequate Se intake according to European Food Safety Authority (EFSA) was set up to 70 μg Se per day for adults [75]. Absorption of Se of both origins, organic and inorganic, takes place in the small intestine. It is required for the synthesis of selenoproteins, regulatory proteins that are distributed to various tissues to render important biological functions. Due to their extensive antioxidant activity, the major role of selenoproteins including glutathione peroxidase (GPX), thioredoxin reductase (TrxR), and iodothyronine deiodinases (IDD), is to prevent oxidative injury of the cell. Thus, Se with its antioxidant properties belongs to important trace elements for humans and is suggested as a dietary supplement for health improvement [76]. The use of Se as a detoxifying element for heavy metals, including Cd, detoxification and its protection mechanism against Cd toxicity has been recently extensively reviewed by [77,78]. Both studies described substantial positive effects of Se, regardless of its form, against Cd toxicity primarily due to its ability to boost the antioxidant defense capacity of the organism. However, they also depicted the possible toxic effects of Se excessive intake (3.2–6.6 mg/day) associated mainly with dermatologic and neurologic symptoms; hence, the safe use of Se in the Cd detoxifying therapy still requires intensive clinical trials.

Numerous scientific laboratories confirmed the positive effect of Se against Cd toxicity using various model systems. The impact of Cd on the trace elements and amino acid profiles in the chicken pectoral muscles has been analyzed by [79]. Inductively coupled plasma mass spectrometry (ICP-MS) analyses revealed that under CdCl_2_ (150 mg/kg for 90 days) exposure levels of lead (Pb), mercury (Hg), aluminum (Al), and lithium (Li) ions increased, while the content of Se (sodium selenite, Na_2_SeO_3_), iron (Fe), and chromium (Cr) decreased. Moreover, Cd-induced disorder of essential amino acids as the levels of valine (Val), leucine (Leu), arginine (Arg), and proline (Pro) significantly decreased which was determined by L-8900 automatic amino acid analyzer. These adverse effects caused by Cd were significantly reversed by the use of Se (0.2 mg/kg of sodium selenite, Na_2_SeO_3_ resulting in 2 mg/kg of Se in the diet) supplementation. The avian leghorn male hepatoma (LMH) cells subjected to CdCl_2_ (2.5 μM for 24 h) suffered from marked disruption of Ca^2+^ homeostasis, alteration of the cadherin (CNX)/calreticulin (CRT) cycle, Cd triggered ER stress and autophagy leading to cell death. It has been shown that Se (either 1.25 or 2.5 μM of Na_2_SeO_3_) enhanced the expression of the components of the calmodulin (CaM)/calmodulin kinase IV (CaMK-IV) signaling pathway and thus mitigated Cd-induced intracellular Ca^2+^ imbalance. The authors concluded that the Se-mediated regulation of Ca^2+^ homeostasis protected LMH cells against Cd-mediated hepatotoxicity as it reduced Cd-elicited crosstalk between autophagy and ER stress [80]. In two independent studies, the chicken liver was used to investigate the toxic effect caused by Cd administration to the standard food (150 mg/kg of CdCl_2_ for 90 or 120 days, respectively), and the protective effect of either inorganic (sodium selenite, Se, 2 mg/kg) or organic (selenium yeast, SeY, 0.5 mg/kg) form of Se, respectively. Cong et al. (2019) [81] detected a Cd-mediated increase in the MDA, DPC (DNA and protein crosslink), and PCO (protein carbonyl) content, while the contents of the cytochrome CYP450, b5, and glutathione (GSH) and the activities of aminopyrine-N-demethylase (AND), erythromycin-N-demethylase (ERND), aniline 4-hydroxylase (AH), NADPH-cytochrome C reductase (CR), decreased. The data indicate that the effect of Cd-induced oxidative stress is related to CYP450 in chickens. Liver intoxication by Cd was, however, largely improved by supplementation of sodium selenite (Se). Similarly, the study by Wang et al. (2020) [82] revealed that Cd addition impaired activities of SOD, GPx, and CAT, elevated levels of MDA, caused liver necrosis, increased expression of the MLKL, Rip1, RIP3, ERK, JNK, and P38 mRNA, and decreased expression of caspase8, while SeY supplementation, to a large extent, restored the Cd-induced alterations. According to this study, Cd mediates chicken liver necroptosis through enhancement of oxidative stress and activation of the MAPK signaling pathway, while SeY prevents this kind of Cd-mediated injury through the reduction of oxidative stress and by the MAPK pathway downregulation. Slightly different results concerning the inorganic and organic forms of Se supplementation were observed by Lynch et al. (2017) [83]. Authors in their study used 0.4 ppm of Se in the cell culture medium in either two organic forms of selenium, selenomethionine (Se-M) or Se-yeast (SeY), and two forms of inorganic selenium, sodium selenite (Se-Ni) or sodium selenate (Se-Na) pre-incubated 48 h prior to Cd intoxication. The effects of such pre-treatment on cell viability and DNA damage following Cd exposure in concentrations of 0.5, 0.7, or 1 ppm CdCl_2_ for 24 h, were evaluated in porcine jejunal epithelial cells (IPEC-J2) incubated in the growth medium containing porcine serum to mimic the porcine gut epithelium. Both organic Se species, when used at the EFSA guideline levels, protected the IPEC-J2 model system against Cd-induced DNA damage and cell death. However, the inorganic Se-Ni and Se-Na did not show any protective effects, and in addition, they even enhanced the negative effects of Cd. This suggests that the porcine gut integrity might be protected from Cd-induced damage only by the use of organo-selenium supplementation.

As it has been shown that Se is also beneficial for plant growth, its impact on Cd toxicity has been evaluated in tall fescue seedlings. Plants exposed to Cd (30 mg/L of CdSO_4_ for 7 days) displayed physiological toxicity symptoms including leaf yellowing, plant height, and root length decrease. The MDA content and electrolyte leakage (EL) increased, while the antioxidant enzyme activities, photosynthetic efficiency, chlorophyll, and soluble protein content, decreased. Moreover, Cd exposure inhibited expression of the antioxidant system-related genes such as chloroplastic Cu/Zn-SOD, cytoplasmic Cu/Zn-SOD, GPx, and ascorbate peroxidase (APX), but not the GR, and photosynthesis-related genes (psbB and psbC), except for psbA. Application of Se (0.1 mg/L, Na_2_SeO_3_ dissolved in water) led to enhancement of the chlorophyll and soluble protein content, CAT, and SOD activities. Se supplementation decreased EL and MDA content and increased the expression of psbA, psbB, psbC, Chl Cu/Zn-SOD, Cyt Cu/Zn-SOD, GPx, and APX. Se thus mitigated Cd-mediated impairments of tall fescue through the improvement of the antioxidant capacity and photosynthesis activity [84]. Consistent with this, a study by Auobi Amirabad et al. (2020) [85] proved Se (2, 4, or 8 mg/L of Na_2_SeO_3_ 6 days after Cd exposure) to serve as Cd (5 and 10 mg/L of CdSO_4_ for 30 days) antagonist in radish plants, as its application to the growth substrate considerably improved Cd-impaired biomass acquisition, enhanced chlorophyll biosynthesis, and increased uptake of essential micronutrients. Se decreased Cd uptake and transport within the radish plant and upregulated the activation of the enzymatic antioxidant protective system. Strikingly, foliar application of Se (10, 20, or 40 mg/L of Na_2_SeO_4_ three times at the tillering, elongating, and heading) on wheat leaves grown in Cd-contaminated soil (0.3 mg/kg soil of CdCl_2_ for 6 months) significantly enhanced the photosynthesis, tissue biomass, and antioxidant enzyme activity, suggesting that foliar spraying could be used as a cost-effective strategy to alleviate Cd toxicity [86].

Altogether, these studies strongly suggest the Se supplementation, predominantly the organic form of Se, to enhance the tolerance of the organism to Cd-mediated stress.

#### 3.2.2. Zinc

Zinc (Zn) is an essential micronutrient ubiquitously found in the body in the amount of approximately 2–3 g in adults. Zn is primarily found in the muscle and bone (85%), skin, and liver (11%), the rest is found throughout the body in different tissues. The everyday refilling of about 0.1% of the overall content of Zn in the organism is regulated through more than three dozen Zn transport regulatory proteins that include the 14 members of the ZRT/IRT-like protein (ZIP) family responsible for the increase in intracellular Zn amounts and the 10 members of the zinc transporter (ZnT) family that regulate the decrease in intracellular Zn [87]. Zinc is an essential element as the 3000 known Zn proteins are involved in myriad physiological processes including the regulation of the innate and adaptive immune responses, growth, tissue maintenance, and wound healing. Zn homeostasis is crucial for sustaining the proper immune function of the organism as it regulates proinflammatory responses of the organism through targeting the transcription factor NF-κB (Nuclear Factor Kappa B), regulates inflammatory cytokines, and controls oxidative stress. Although Zn toxicity is rare, its intake dramatically exceeding the recommended dietary allowance (RDA) of 15 mg per day may induce symptoms of anemia and neutropenia resulting in fatigue, epigastric pain, or nausea [88,89]. However, malnutrition leads to zinc deficiency resulting in various health complications such as impaired wound healing, altered immune responses, or skin rash. As the association between zinc status and a normal state of health is an undeniable fact, the additional Zn supplementation might be efficacious to prevent certain conditions as for example the abiotic contamination caused by Cd [90]. Numerous studies have recognized Zn as a preserving element against Cd toxicity. Although Zn and Cd have similar chemical structures, Zn, when not overdosed, has not been shown to have adverse side effects on the organism. However, due to their similar structure and charges, Zn hinders Cd uptake by the cell. Commonly, the mechanisms underlying the Cd toxicity prevention by Zn might be summarized in the following activities: direct competition of the two metals, Zn-derived MT induction, and Zn-triggered redox homeostasis. Current knowledge related to the role of Zn in Cd toxicity reduction has been recently reviewed by Yu et al. (2021) [91].

A variety of cellular, animal, and plant model systems have been used to investigate the effect of Zn supplementation on Cd-mediated impairments. The experiments by Branca et al. (2018) [92] carried out on SH-SY5Y catecholaminergic neuroblastoma cell line demonstrate the toxic activity of Cd (10 μM CdCl_2_ for 24 h) causing a decrease in the cell viability, oxidative stress, and endoplasmic reticulum (ER) stress triggering neuronal sprouting. The addition of Zn (50 μM ZnCl_2_), as well as Se (100 nM Na_2_SeO_3_), showed that both elements were able to attenuate the Cd-induced neurotoxicity. In a different cellular model, the Madin–Darby bovine kidney (MDBK) epithelial cells were used to demonstrate the protective role of Zn in counteracting Cd (10 or 50 μM CdCl_2_ for 3, 6, 12, or 24 h) toxicity. The ability of Zn (10 or 50 μM ZnCl_2_) to alleviate Cd uptake by the cell, Cd-mediated apoptotic cell death, mitochondrial damage, and oxidative stress, designates Zn supplementation as a protection element for livestock against excessive Cd accumulation [93]. Gene expression analyses by Pan et al. (2017) [94] in yeast *Saccharomyces cerevisiae* exposed to Cd (40, 80, 160, 320 μM CdSO_4_ for 2 h) or Cd plus Zn (40, 80, 160, 320, 640 μM ZnSO_4_) revealed that Cd-induced changes in the expression of 912 genes, while Cd plus Zn induced expression changes in 627 genes. Zn treatment to Cd-stressed cells entirely reversed the expression of 48.7% genes from the 92.1% efficiently reversed genes from all genes differentially expressed by Cd. According to the gene expression results, the multidirectional mechanism of cell defense by Zn treatment results from Zn-triggered inhibition of expression of genes associated with Cd-mediated oxidative stress, Zn-mediated preservation of expression of genes involved in ion homeostasis, and mitochondrial membrane potential (MMP) maintenance, and Zn-mediated inhibition of the expression of the Cd-altered expression of genes involved in sulfur and GSH metabolism. Moreover, Zn reduced the intracellular level of Cd and inhibited the synthesis alterations of Cd-induced ribosomal proteins, S-containing amino acids, S-rich proteins, and antioxidant enzymes. Although not all the Cd-altered gene expressions, including genes involved in amino acid metabolism, nitrogen metabolism, oxidative phosphorylation, or ubiquitin-mediated proteolysis, were corrected by Zn supply, Zn represents substantial protection against Cd toxicity. An interesting study by Ben Mimouna (2018) [95] deals with the Cd-induced neurotoxicity on rat pups from mothers receiving Cd (50 mg/L CdCl_2_), Zn (60 mg/L ZnCl_2_), or Zn plus Cd in drinking water during gestation and lactation and the impact of Zn on Cd toxicity. The hippocampal volume measurement revealed that the indirect Cd exposure significantly altered and decreased the volume of the CA1, CA3 pyramidal cell layer, and the dentate gyrus. Moreover, the superoxide dismutase (SOD) activity and the metallothionein (MT) level significantly increased in rat litters. Zn co-treatment largely corrected alterations caused by Cd exposure highlighting its protective effect against Cd-mediated impairment of the hippocampus a crucial structure implicated in learning and memory processes.

As Cd is known to cause reproductive toxicity, its impact, and probable benefits of Zn on morphological, physiological, and antioxidant parameters on the freshwater crab *Sinopotamon henanense* and rat testis, were analyzed. Exposure of Cd (0.05, 0.1, and 0.5 mg/L CdCl_2_ for 14 days, or 1 mg/kg/day CdCl_2_ by i.p. injection for 21 days, respectively), reduced sperm count and motility, caused histological damage, morphological lesions, decreased the relative testis weight (RTW), increased SOD, CAT, GPx activity and MDA levels. In both experimental animal models, Zn (0.1 and 1 mg/L ZnSO_4_ or 0.5 and 1.5 mg/kg/day ZnCl_2_, respectively), treatment prevented or reversed Cd-induced testicular toxicity mainly due to improvement of the antioxidant status [96,97]. Moreover, rat testicular development under Cd stress and the effect of Zn was tested in male litters from mothers receiving Cd (50 mg/L CdCl_2_ in drinking water), Zn (60 mg/L ZnCl_2_ in drinking water), or Zn plus Cd during gestation, or gestation and lactation. Cd-mediated disruption of Zn metabolism resulting in maternal hypozincemia during gestation caused Zn deficiency in the fetus which was even more pronounced during lactation. Depletion of Zn in turn led to progressive Cd accumulation at postnatal day (PND) 12 and PND21 consequently resulting in the formation of abnormal seminiferous tubules followed by the decrease in testis weight and plasmatic testosterone concentration at PND21 and PND35. Interestingly, Zn treatment considerably protected male rats from Cd-induced testicular toxicity, suggesting that the toxic effect of Cd is mediated through the disruption of prenatal Zn metabolism, which is established in mothers during pregnancy [98]. An intriguing study focused on the analyses of the impact of Zn (100, 200, and 400 μg/g of food) on Cd (44 μg/g of food, a sublethal Cd concentration) adapted beet armyworm *Spodoptera exigua* selected after long-term Cd exposure for 135 generations was performed by Tarnawska et al. (2019) [99]. The life history traits represented by the duration of L4 and L5 stages, cellular DNA damage, and biochemical parameters (ADP/ATP ratio and ATP and HSP70 concentrations) were determined. The analyzed larval stages of Zn supplemented larvae were significantly prolonged, while cellular and biochemical indicators appeared to be corrected as compared to the group of Zn untreated insects. The authors of the study suspect that Zn supply contributes to the protection of *Spodoptera exigua* against Cd intoxication, however at the cost of growth rate.

Exogenously applied Zn on the leaves or to the soil with the aim to reduce Cd toxicity has also been used in plants to reduce the negative effect of Cd on plants. Zn mitigates Cd toxicity primarily through the increase in the antioxidant capacity and by regulation of the Cd transport leading to the increase in plant growth, and photosynthesis (reviewed by Rizwan et al. (2019) [100]). It has been reported that the Zn treatment (either as foliar spray application of 0.2% ZnSO_4_, or soil supplemented by 41.2 mg/kg ZnSO_4_) on plants planted in pots containing 2.5 kg of soil from the location near a coal mine, naturally contaminated by Cd, significantly downregulates the influx transporter gene TaNramp5 and upregulated the efflux transporters TaTM20 and TaHMA3 in wheat, thus limiting Cd accumulation. Additionally, the leaf TaHMA2 expression was downregulated upon foliar Zn applications causing a reduction in Cd translocation from root to shoots. Soil Zn application downregulates expression of the TaLCT1 gene in roots, thereby reducing the root Cd concentration. Moreover, this leads to the efficient reduction of Cd in grains, hence ensuring concomitant food and yield safety [101].

As the widespread use of nanoparticles in food and pharmaceutical products is a conquest of the modern age, Sharifan et al. (2020) [102] investigated the impact of nano-zinc oxide (100 mg/L in deionized water) against heavy metal toxicity (1 mg/L of CdSO_4_ in combination with 100 mg/L of Pb(NO_3_)_2_ for 2 weeks) toxicity in leafy green species spinach (*Spinaciae oleracea*), parsley (*Petroselinum sativum*) and cilantro (*Coriandrum sativum*), and Hejazy et al. (2017) [103] analyzed the effect of the metal Zn (10 mg/kg b.w. ZnCl_2_ in drinking water for 28 days), and nano-zinc particles (3, 10, and 100 mg/kg b.w. in the daily gavage) to Cd-induced toxicity (2.5–5 mg/kg b.w. of CdCl_2_ in drinking water for 28 days) in rats. Nano-zinc oxide particles elevated the copper and iron content in shoots resulting in the limited absorption of Cd by roots thus protecting three analyzed leafy green species from Cd toxicity.

Surprisingly, in rats, despite the clear evidence of the metal Zn protection from Cd toxicity, nano-Zn particles (15, 20, and 30 nm) are not suitable for protection against cadmium intoxication. Blood analyses of animals treated with nano-Zn particles displayed significantly increased hematocrit (HCT) and significantly decreased blood coagulation time. Moreover, AST, ALT, triglyceride, total cholesterol, LDL, and free fatty acids increased markedly after Cd- and nano-zinc addition compared with the control group of rats, while metal Zn alleviated Cd-induced toxicity of the organism. This implies that nano-Zn might be used for plant protection from Cd impairment while metal Zn is more suitable for animal and probably also human protection.

#### 3.2.3. Calcium

Calcium (Ca) as an essential macronutrient involved in various physiological processes, including growth and development, cell division, cytoplasmic streaming, and intracellular signal transduction could be used as an exogenous additive to protect organisms against Cd stress due to its chemical similarity with Cd. The use of Ca supplementation for Cd stress elimination in plants has been extensively reviewed by Huang et al. (2017) [104] providing evidence that plants with elevated Ca levels possess improved resistance to Cd-derived alterations. The toxic effect of Cd has been widely studied in different plant species. Seedlings of *Matricaria chamomilla* L. hydroponically cultured under 0, 120, and 180 μM CdCl_2_ stress conditions for 2 weeks were treated with 0, 0.1, 1, and 5 mM CaCl_2_ in the study of Farzadfar et al. (2013) [105]. The addition of Ca significantly ameliorated Cd-induced enhancement of MDA content, decreased antioxidant enzyme activity, and accumulation of ROS in the plants. Additionally, Ca helped to increase the growth parameters resulting in biomass accumulation and reduced the influx of Cd ions. In the study of Li et al. (2016) [106] Arabidopsis seedlings exposed to Cd (50 μM of CdCl_2_ for 5 days) displayed increased oxidative stress as determined by enhanced H_2_O_2_ content and lipid peroxidation. Furthermore, the distribution and level of auxin was altered and the primary root (PR) length was decreased, while the number of lateral roots (LR) increased. Application of Ca (3 mM CaCl_2_ for 5 days) not only led to oxidative stress reduction but also restored the auxin homeostasis in Arabidopsis seedlings under Cd exposure thus alleviating the Cd-induced root growth inhibition. Soil contamination by Cd represents a significant risk not only to plant production but also to human health. Two independent studies analyzed the effect of Ca (2.5 and 5 mg/kg soil of CaCl_2_, or 0.05, 0.5, ad 5 mM Ca(NO_3_)_2_ in the basal salt medium (BMS) background solution, respectively, for either 3 days or until tillering or ripening stage) in the regulation of Cd (50 and 100 mg/kg soil of CdCl_2_, or 50 μM Cd(NO_3_)_2_ in the background of BMS, respectively), intake and translocation, Cd-mediated oxidative damage, the yield and related components in rice grown on Cd-contaminated soil [107,108]. The addition of 5 mM Ca to the soil contaminated with Cd led to OsNRAMP1 and OsNRAMP5 transporter genes downregulation resulting in significantly reduced Cd uptake by roots, while upregulation of OsHMA2 transporter gene and downregulation of OsHMA3 the heavy metal ATPase increased the root-to-shoot Cd translocation. Despite the Ca-mediated increase in the Cd content in the aboveground plant tissues, this double-edge role of Ca in Cd metabolism ultimately resulted in the decrease in Cd content in rice grains [107]. Moreover, Ca treatment (2.5 and 5.0 mg/kg soil) mitigated Cd-derived oxidative stress, enhanced activities of enzymatic antioxidants (SOD, POD, CAT), increased the number of carotenoids and photosynthetic pigments, improved the proline, soluble protein, and soluble sugar contents in rice, altogether leading to the yield gain arising from the increased number of panicles, spikelet per panicle, seed setting rate, 1000 grain weight, and grain yield. Furthermore, Cd accumulation in roots, stems, leaves, and grains was significantly reduced with Ca amendment, suggesting that Ca-based supplementation has the potential to produce low Cd rice grains and to ameliorate the toxic effect of Cd in crops grown in Cd-contaminated soils [108].

An attractive experiment was performed with chickpea (*Cicer arietinum* L.) seeds exposed to 200 μM CdCl_2_ for 6 days concomitantly treated with either 100 mM CaCl_2_ or 100 μM EGTA (ethylene glycol tetraacetic acid). Such treatment led to a 2.75- and 1.75-fold increase in Cd-reduced thioredoxin (Trx) and thioredoxin reductase (NTR) activities, reduction of H_2_O_2_ content, and modulation of the ferredoxin (Fdx) activity to a control level. Moreover, the addition of Ca/EGTA counteracted the detrimental impact of Cd on the gene expression and activity of Cu/Zn-SOD and stimulated the activities of CAT and APX hence protecting plants from Cd contamination [109]. Protective effect of Ca (100 and 1000 μM of CaCl_2_ for 24 h) against Cd toxicity (exposure of 10 and 100 μM CdCl_2_) was also detected in lichen *Hypogymnia physodes* and confirmed by Ca-derived suppression of Cd accumulation, a decrease in ROS generation and lipid peroxidation, normalization of thiol, glutathione, and ascorbic acid levels. The results indicate that the ameliorative response of Ca under Cd exposure in lichens is comparable with its effect in plants [110]. It is worth noting that in addition to plants or lichens, Ca protection against Cd toxicity was experimentally documented in single-celled microorganisms, yeast *Saccharomyces cerevisiae* [111], and marine bacterium *Bacillus* sp. 98 [112]. *S. cerevisiae* cells exposed to Cd (50, 100, 200, and 500 μM Cd^2+^ for up to 60 min, or 2–3 days, depending on the experimental setup) responded with a dramatic increase in cytosolic Ca released from the vacuole through the Yvc1p channel, which together with external Ca (10 mM Ca^2+^) that reached cells via the Cch1p/Mid1p channel-mediated cell adaptation to Cd stress [108]. Bacterial cells upon Ca (5, 10, 15, 20, 25, or 30 mM Ca^2+^) supplementation reduced Cd (0.2, 0.4, 0.6, 0.8, 1, 1.5, or 4 mM Cd^2+^)-induced excess of intracellular nitric oxide (NO) production through reduction of NO synthase expression and enhancement of NO dioxygenase expression. Moreover, the self-protection mechanism of bacteria in Cd contaminated environment upon the presence of extra Ca additionally involves an increase in the expression of proteins associated with iron uptake resulting in the enhancement of iron acquisition [112].

Furthermore, the protective effect of waterborne or dietary Ca (30 and 60 mg/g food of CaCl_2_, or 20 and 60 mg/kg diet CaCO_3_, or 0.2 mM low Ca^2+^ and 0.8 mM high Ca^2+^ freshwater concentration, respectively), against Cd (50 μg/L Cd(NO_3_)_2_ for 7 days as acute waterborne exposure, or 3 μg/L waterborne and 500 mg/kg diet Cd(NO_3_)_2_ for 28 days, or ambient Cd^2+^ 10 μg/L artificial freshwater and dietary Cd^2+^ 10 μg/fish/day for 2, 4, 14, and 35 days, respectively), toxicity has been detected in aquatic model animals juvenile rainbow trout (*Oncorhynchus mykiss*) or Mozambique tilapia (*Oreochromis mossambicus*) showing the more pronounced effect of the dietary Ca. Diet supplied by Ca causes short-term whole-body reduction of waterborne Ca and Cd uptake by >50% which in turn significantly decreases the chronic accumulation of Cd in targeted tissues, suggesting that the transport mechanism for Ca and Cd intake uses common pathway(s) in the gill and gut. Moreover, Ca supplementation protected the structure and morphology of corpuscles of Stannius and bony tissue damage caused by Cd [113,114,115].

Another confirmation of the positive effect of Ca against Cd stress results from studies on mammals. Element analyses by atomic absorption spectrometry were performed in the liver, kidney, brain, and carcass (body acquitted of organs and skin) tissues obtained from rat pups subjected to either oral or parenteral Cd (0.5 mg/kg body weight of CdCl_2_ for 10 days) exposure and fed by Ca supplemented milk (1%, 3%, and 6% of CaHPO_4_). Calcium supplementation was able to decrease Cd content elevated upon oral submission, while levels of essential elements remained unaffected. However, Ca supply as designed in this analysis was not sufficient to influence the consequences of parenterally administered Cd, suggesting that during the suckling period, Ca excess might help to reduce absorption and retention of orally exposed Cd without affecting trace elements content in the tissues [116]. In addition, the ability of Ca supplementation (0.4% Ca^2+^) to reduce Cd-mediated bone damage was analyzed in young female rats exposed to Cd (1, 5, and 50 mg CdCl_2_/kg diet for 90 days). Cd-mediated bone formation and quality impairment were significantly restored by Ca supplementation. Ca supply increased bone biomechanics, bone formation marker level in the serum, and expression of osteogenic gene markers, while serum Klotho level, fibroblast growth factor 23/Klotho-associated gene expression, and bone microstructure damage, decreased [117]. Moreover, administration of Ca (100 mg/kg of CaCO_3_ orally administered five times per week) alone or together with vitamin D (600 UI/kg three times per week), as well as the use of calcimimetic compound NPS R-467 (10 μM injected in the tail vein) alleviate Cd-induced hepatotoxicity in rats and nephrotoxicity in mice, respectively. Hepatic tissue exposed to CdCl_2_ (44 mg/L in drinking water for 4 weeks) disposed markedly of elevated pro-oxidants including MDA, H_2_O_2_, and protein carbonyls. The actual level of inflammatory cytokines such as interleukin (IL) 1β, IL-6, IL17A, and tumor necrosis factor-α increased, while the anti-inflammatory IL-10, IL-22 markers, and antioxidants GSH, GPx, and CAT activities, decreased. Additionally, Cd exposure induced hypovitaminosis D, depression of Ca levels in hepatic tissue, altered expression of Ca-binding proteins (CAM/CAMKIIA/S100A1/S100B), store-operated (RyR1/ITPR1) channels, and vitamin D-metabolizing enzymes (Cyp2R1/Cyp27a1/cyp24a1) in the rat liver [118]. Similarly, mice exposed to Cd (10, 100, 1000 ppm CdCl_2_ in the diet for 28 days) displayed kidney damage manifested by glomerular atrophy, renal proximal tubule injury, elevated MDA level, enhanced amount of urine protein, and upregulated expression of kidney injury molecule 1 (KIM-1). Furthermore, long-term Cd exposition caused inhibition of autophagy flux triggering kidney apoptosis and damage [119]. Co-supplementation of Ca and vitamin D largely reduced Cd content in the serum and tissues, and inflammatory and oxidative markers in the liver suggest that concomitant treatment with vitamin D and Ca substantially protects organisms against Cd-induced hepatotoxicity. Strikingly, activation of CaSR with the use of NPS R-467 results in the revitalization of Cd-inhibited autophagy flux leading to reduction of kidney apoptosis and damage. Hence, calcimimetic compound NPS R-467 as well as Ca/vitamin D treatment might serve as suitable strategies to prevent Cd-mediated nephrotoxicity and hepatotoxicity, which might in turn help to treat Cd-derived clinical chronic kidney or liver disease [118,119].

#### 3.2.4. Silicon, Magnesium, Manganese

The effect of silicon (Si), magnesium (Mg) and manganese (Mn) on Cd toxicity has also been investigated.

Mitigation of the CdCl_2_ (5 mg/L for 7 days)-induced toxicity with the use of Si (1 mM/L of Na_2_SiO_3_) and/or Se (1 μM/L Na_2_SeO_3_) supplementation was analyzed in hydroponically grown ramie *Boehmeria nivea* (L.) *Gaud*. Application of not only Se but also Si decreased the content and root to aboveground parts translocation of Cd in plants. Moreover, the treatment resulted in the stimulation of antioxidant enzyme activity including SOD, guaiacol peroxidase (POD), and APX leading to the decrease in total ROS, MDA, and H_2_O_2_ content in ramie leaves. Additionally, Si and Se separately or in combination protected plants from Cd stress through elevation of the content of nonenzymatic antioxidants such as glutathione, ascorbate, and vitamin E [120]. The authors [121] came to similar conclusions in their study investigating the possible alleviation of Cd toxicity in rice (*Oryza sativa* L.) by the use of Si and Se nanoparticles (NPs) in concentrations of 5, 10, and 20 mg/L, separately or in combination. Authors of the study planted rice in heavy metal contaminated soil, due to excessive use of phosphate fertilizers the soil contained 0.84 mg/kg Cd^2+^ ions. They concluded that foliar application of Si and Se-Nps reduces the content of Cd and phytic acid in brown rice which improves the nutritional quality of rice grains. Interestingly, Cd toxicity toward *Phytolacca acinosa* Roxb cultured under hydroponic conditions is also significantly reduced by manganese (0.5, 1, 1.5, 2, 2.5, 3, 5, 6, 10, or 12 mM Mn^2+^) treatment and is largely controlled by the manganese/cadmium molar ratio. The defense against Cd (exposure to 50, 100, 200 μM of Cd^2+^ for 17 days) toxicity in *P. acinosa* seedlings is ensured by elevated Mn distribution in shoots at low levels of solution Mn/Cd molar ratio (SMCR) (e.g., 0 and 10), at high levels of SMCR (e.g., 50 and 60) the plant protection occurs through significantly reduced lipid peroxidation and plant water loss, and increased photosynthesis arising from the antagonism between Mn and Cd in the plant [122]. Moreover, Mn has been reported to prevent acute Cd (single dose of s.c. injection of 7 mg/kg b.w. CdCl_2_) intoxication in mice tissues due to its antioxidant properties. Manganese (i.p. injection of a single dose of 20 mg/kg b.w. MgCl_2_ 24 h prior to Cd exposure) treatment prevented Cd-enhanced GSH-Px activity and LP (lipid peroxidation), Cd-mediated GSH depletion, and significantly attenuated CAT activity. Additionally, Mn supplementation of Cd-treated mice led to a significant decrease in Cd content in the kidneys and testes, while Cd content in the liver increased as a consequence of a diverse metallothionein induction in individual organs. Manganese-mediated protection against Cd toxicity also depends on its ability to limit Cd-derived alterations of Ca homeostasis [123]. In addition, reduced Cd intake (cell exposure to 0.5–2 μM CdCl_2_ for 24 h) upon Mn supplementation (2, 4, 8, 16, 32, and 64 μM MnCl_2_) associated with the inhibition of the Cd-promoted sustained ERK activity has been shown by Martin et al. (2006) [124] in different cell types. Reported results indicate that Mn could be considered as an effective competitor for cadmium toxicity.

Furthermore, experiments with animal model systems revealed the protective role of magnesium (Mg) against Cd stress. Analyses of the blood plasma of rats exposed to Cd showed dramatic alterations in the oxidative status of the animal. The negative effect of orally or intraperitoneally administered single dose of Cd (30 mg/kg b.w. by orogastric tube or 1.5 mg/kg b.w. by i.p. injection of CdCl_2_, respectively), was manifested by the decrease in SOD activity, increased amount of superoxide anion, affected total oxidative status, the elevation of advanced oxidation protein products, and increased content of MDA. Oral or intraperitoneal pre-treatment of rats with Mg (50 mg/kg b.w. or 3 mg/kg b.w. of Mg(CH_3_COO)_2_ 1 h or 10 min prior to CdCl_2_ treatment, respectively), substantially prevented Cd-triggered changes in the examined parameters [125]. Consistent with this, rats receiving 1 mg/kg CdCl_2_ together with 0.5 or 1.5 mg/kg MgCl_2_ by i.p. injection for 21 days, were protected against Cd-induced nephrotoxicity. Magnesium treatment reversed Cd-triggered kidney damage, Cd-enhanced kidney MDA content and serum sodium, potassium, and urea levels, and Cd-reduced creatinine, and protein levels [126]. Moreover, [127] confirmed the positive impact of Mg against Cd-induced hepatotoxicity with the use of isolated perfused rat liver (IPRL) model system. Liver exposure to Cd (15 μM of CdCl_2_ for 30, 60, and 90 min) decreased glutathione level, enhanced MDA content and aminotransferase activity in IPRL model, while Mg (1.2 mM of MgSO_4_) cotreatment reduced the toxicity of Cd. Hence, these results provide evidence that Mg supplementation may have protective effects against Cd toxicity.

The toxic effect of Cd on the tested systems and its alleviation by mineral elements supplementation is summarized in Table 2.

### 3.3. Bioactive Substances

#### 3.3.1. Polyphenols, Phenols, and Phenolic Acids

Phenolic compounds are plant-derived secondary metabolites typically known for their eminent antioxidant properties. The role and metabolism of phenolic compounds in the human organism is still the subject of intense study. They are characterized by highly diverse chemical structures, occurring in free or bound form with compounds including saccharides and organic acids. In general, phenolic compounds according to their structure can be divided into three major subgroups: (i) phenyl-carboxyl acids—derivatives of benzoic acid, e.g., protocatechuic acid and gallic acid; (ii) phenyl-propene acids—derivatives of cinnamic acid, e.g., ferulic acid, vanillic acid or caffeic acid; (iii) flavonoids, the most known and abundant group [128].

##### Flavonoids

Flavonoids, usually found in foods of plant origin, have been proposed as protective substances against Cd-derived stress. According to their chemical structure, they can be divided into the following subgroups: flavanones, flavones, flavonols, flavan-3-ols, anthocyanins, and isoflavones. The primary mechanism of flavonoids to reduce Cd toxicity has been suggested through their ability to improve the antioxidant capacity of the organism, Cd sequestration resulting in the ionome balance maintenance, and protection of DNA from Cd-induced damage. Moreover, flavonoids are able to suppress inflammation, positively regulate glycometabolism, and promote the secretion of reproductive hormones [129].

Here, we introduce some of the flavonoids described to discriminate Cd-induced impairments.

- *Rutin hydrate*

Oboh et al. (2019) [130] and Abdel-Aleem et al. (2018) [131] investigated the role of the flavonol glycoside rutin hydrate (IUPAC name: 2-(3,4-dihydroxyphenyl)-5,7-dihydroxy-3-[(2S,3R,4S,5S,6R)-3,4,5-trihydroxy-6-[[(2R,3R,4R,5R,6S)-3,4,5-trihydroxy-6-methyloxan-2-yl]oxymethyl]oxan-2-yl]oxychromen-4-one;hydrate) commonly found in plant food against Cd-induced neurotoxicity and cognitive disturbances in rats. The experimental group of rats received by oral administration either 25 and 50, or 100 mg/kg rutin for 14 or 30 days, respectively, in addition to 5 mg/kg CdCl_2_. Apart from the strong antioxidant properties resulting in the decrease in Cd stress and residual Cd ions, rutin alleviated Cd-mediated cognitive impairments by restriction of the ERK1/2 and JNK apoptotic pathways, and by inhibition of PTEN-derived regulation of mTOR survival pathway activation in the rat brain. Moreover, rutin treatment decreased ectonucleotidases, adenosine deaminase (ADA), and MAO activities, while thiol levels increased. Thus, rutin is capable to restore brain damage and memory dysfunction caused by Cd via improvement of the Cd-altered activity of the purinergic and monoaminergic regulatory enzymes.

- *Chrysin*

Chrysin (IUPAC name: 5,7-dihydroxy-2-phenylchromen-4-one), a flavonoid found in bee products, has been shown to dispose of strong antioxidant, anti-inflammatory, and hepatoprotective properties, is due to its therapeutic activities eligible to be considered as a food supplement. In the study of Beyrami et al. (2020) [132] the authors used 2.5 and 5 mg/kg body weight of nanoliposome-loaded chrysin (NLC) to investigate its possible protective role against Cd (2 mg/kg b.w. Cd^2+^ in drinking water every day within 30 consecutive days) toxicity in mice. Cadmium-induced hepatic stress, liver enzyme enhancement, morphological changes in jejunum, and food intake alteration were restored upon NCL supplementation. Hence, the NCL can be suggested as a food additive, to eliminate Cd-mediated alterations in mice.

- *Diosmin*

Diosmin (IUPAC name: 5-hydroxy-2-(3-hydroxy-4-methoxyphenyl)-7-[(2S,3R,4S,5S,6R)-3,4,5-trihydroxy-6-[[(2R,3R,4R,5R,6S)-3,4,5-trihydroxy-6-methyloxan-2-yl]oxymethyl]oxan-2-yl]oxychromen-4-one) a flavonoid found predominantly in the pericarp of citruses, characterized as strong antioxidant, possesses among others also anti-tumor, anti-inflammatory, anti-hyperglycemic, anti-hyperlipidemic, anti-hypertensive, and anti-mutagenic properties. Similar to chrysin, diosmin (100 mg/kg b.w. by oral administration) showed protective responses against Cd-induced hepatotoxicity in rats exposed to CdCl_2_ in a dose of 200 ppm via drinking water for 30 days. Liver enzyme levels and activities, antioxidant parameters, histopathological parameters, and body weight altered by Cd, were markedly restored in animals receiving diosmin (100 mg/kg/bw) supplementation [133].

- *Quercetin*

Quercetin (IUPAC name: 2-(3,4-dihydroxyphenyl)-3,5,7-trihydroxychromen-4-one) and its derivatives are the most widely distributed plant flavonoids ubiquitously found in herbs, vegetables, and fruits. Various kinds of quercetin-triggered physiological activities, such as antioxidant, anti-inflammatory, or immune regulatory, have been described. Due to its undoubted ability in scavenging free radicals, it acts as a strong antioxidant with potential feasibility in the prevention of cancer, diabetic nephropathy, or renal fibrosis. The protective effect of quercetin against Cd toxicity has been studied. Cadmium (0.4 mg/kg b.w. CdCl_2_) intraperitoneally injected in mice for 3 days induced characteristic autophagosome formation in the kidney, increased the LC3-II/β-actin ratio, dramatically enhanced ROS level, and MDA content, while the total antioxidant capacity decreased. Autophagy caused by Cd-induced oxidative stress in mouse kidneys was markedly restored by quercetin in concentrations of 5 to 100 mg/kg b.w. with the most significant effect at 25 mg/kg b.w. quercetin [134]. Similarly, rats intraperitoneally treated with Cd (2 mg/kg b.w. CdCl_2_ for 4 weeks) displayed decreased body and relative testicular weight, and pathological changes in testes. In addition to Cd-triggered oxidative stress, the protein expression levels of P62 and LC3-II increased under Cd exposure referring to autophagy. Conversely, oral administration of quercetin (50 mg/kg b.w.) alleviated Cd toxicity by reducing oxidative stress and inhibiting autophagy [135]. Strikingly, primary rat proximal tubular (rPT) cells exposed to 2.5 μM Cd(CH_3_COO)_2_ for 12 h displayed impaired autophagic flux and diminished lysosomal degradation capacity. Quercetin (1 μg/mL) treatment decreased levels of autophagy marker proteins and led to TFEB-dependent recovery of lysosomal function, as quercetin, a recognized mTORC1 inhibitor, enhanced autophagy by promoting TFEB activity. Moreover, Cd-induced lysosomal alkalization caused by v-ATPases inhibition was significantly restored by quercetin treatment [136]. Another study explored the quercetin (10 and 50 mg/kg b.w. by oral gavage) protection of rat kidneys against Cd (4.89 mg/kg b.w. CdCl_2_ in drinking water for 12 weeks) toxicity utilizing metabolomics methods. Metabolomics and histopathology examination of the kidney showed Cd-derived significant changes in 11 analyzed metabolites that were restored by high doses of quercetin. This suggests that Cd-triggered oxidative stress alters lipids, amino acids, and purine metabolism resulting in nephrotoxicity which can be attenuated by quercetin [137]. Moreover, Cd (60 μM of CdCl_2_ exposure at 38.5 °C for 4, 8, and 12 h)-induced oxidative stress in goat sperm during storage impaired sperm motility, survival rates, membrane integrity, and mitochondrial activity that resulted in altered embryo development. Sperm pre-incubation with quercetin (10 μM) protects not only goat sperm but also preimplantation embryos from Cd-derived oxidative stress [138]. Studies on Cd association with memory impairment of the F1–F2 generation in mice were determined by the use of Morris water maze and step-down latency test. The impact of intraperitoneal administration of Cd (1.2 mg/kg/day of CdCl_2_) for 1 week during the gestation period on newborn pups was evaluated. Brain tissue of F1 generation displayed significantly enhanced activity and expression of GST and CAT and showed memory impairment. Concomitant treatment with quercetin (25, 50, and 100 mg/kg b.w.) reversed this effect. In the F2 generation, the results were variable. The study implies that Cd might cause memory impairment through altered antioxidant enzyme activity and gene expression in the brain tissue. Quercetin treatment improves the antioxidant capacity and reduces Cd levels in brain tissue of F1 and F2 generation [139]. Taken together, quercetin protects cells from Cd toxicity predominantly by alleviating Cd-triggered oxidative stress.

- *Hesperetin*

Hesperetin (IUPAC name: (2S)-5,7-dihydroxy-2-(3-hydroxy-4-methoxyphenyl)-2,3-dihydrochromen-4-one), a derivative of hesperidin found in abundance in citrus fruits belongs to flavanone class of flavonoid. Varieties of biological activities of hesperetin have been described. The most prominent positive features of hesperetin belong to its antioxidant property and free radical elimination, anti-cancer and genotoxic activities, modulation of the cardiovascular system, protection of the nervous system, and anti-bacterial and microbial activities [140,141]. Shagirtha et al. (2017) [142] investigated the protective effect of hesperetin against Cd-induced neurotoxicity in rats. Subcutaneous injection of 3 mg/kg b.w. CdCl_2_ daily within 21 days, led to decreased activity of acetylcholinesterase in the brain, oxidative stress manifested by enhanced ROS and carbonylated proteins, while enzymatic (SOD, CAT, DPx) and non-enzymatic antioxidants (GSH, total sulphydryl groups, and vitamin C) were reduced. Moreover, typical apoptotic markers (Bcl2 associated X protein (Bax), cytochrome C, caspase 3 and 9) increased, whereas anti-apoptotic marker (B-cell lymphoma 2 (Bcl2)) in the brain of Cd-exposed rats decreased. In addition, the mitochondrial electron transport chain complexes (I, II, III, and IV) in the rat brain decreased upon Cd exposure. Importantly, hesperetin treatment (40 mg/kg b.w., oral administration) mitigated the Cd-derived oxidative stress and mitochondrial dysfunction and decreased apoptosis in the rat brain.

- *Anthocyanins*

Anthocyanins, colored water-soluble pigments, exist widely in plants and belong to the most abundant flavonoids exceeding 500 different types. A wide variety of biological activities of anthocyanins have been described. These beneficial health effects include antioxidant and antimicrobial activity, anti-inflammation, anti-mutagenesis, induction of differentiation, inhibition of cancer cells proliferation, anti-metastasis, and heavy metal sequestration. Anthocyanins use different biological pathways to protect organism from the negative impact of the environment such as mitogen-activated protein kinase (MAPK) pathway, free-radical scavenging pathway, cyclooxygenase pathway, or inflammatory cytokines signaling [143,144].

Rat exposure to 4 μg/kg b.w. of CdCl_2_ in aqueous solution via stomach tube for 30 days, resulted in Cd accumulation in the liver and kidney, increased concentration of bilirubin and urea in blood serum, enhancement of ALT and AST activity. Co-administration of anthocyanins (10 mg/kg b.w.) protected rat liver and kidney from Cd-induced impairments [145]. The effect of anthocyanin cyanidin-3-O-glucoside (C3G) (500 mg/kg/day in chow) on the endocrine-disrupting effect of Cd was determined in male mice exposed to 5 mg/kg/day of CdCl_2_ via gavage for 10, 20, and 30 days. It has been shown that C3G treatment reversed the Cd-altered levels of gonadotropins, luteinizing hormone (LH), and follicle-stimulating hormone (FSH) in the serum. Additionally, Cd-reduced sex hormone receptor Gnrh1 gene expression was restored by C3G, hence improving the expression of LH and FSH receptors in the testes leading to activation of the signaling pathway related to the synthesis of testosterone. This suggests the protective effect of anthocyanins consumption against Cd-derived male reproductive dysfunction [146].

- *Naringin*

Naringin (IUPAC name: (2S)-7-[(2S,3R,4S,5S,6R)-4,5-dihydroxy-6-(hydroxymethyl)-3-[(2S,3R,4R,5R,6S)-3,4,5-trihydroxy-6-methyloxan-2-yl]oxyoxan-2-yl]oxy-5-hydroxy-2-(4-hydroxyphenyl)-2,3-dihydrochromen-4-one), a polyphenolic compound obtained from citrus plants possesses –OH groups that trigger its antioxidant activity. It has the ability to neutralize peroxyl, superoxide, and hydroxyl radicals in a dose-dependent manner. Naringin treatment is associated with the enhancement of GSH and GPX, GR, SOD, and CAT enzyme activities [147]. Exposure of Cd (50 μM CdCl_2_ for 24 h) to human hepatocellular carcinoma (HepG2) cells induced oxidative stress and altered the antioxidant system, resulting in cytotoxicity. Cell co-treatment with naringin (5 μM) reconstituted redox homeostasis, mitochondrial membrane potential, and reduced apoptosis. Its antioxidant property maintained the endogenous activities of SOD, GST, and CAT, while lipid peroxidation, caspase 3 cleavage, and cytochrome c release decreased. Thus, the protective mechanism of naringin is attributed to its antioxidant potential that prevents Cd-mediated cytotoxicity [148]. Similarly, in human lymphocytes naringin (1 and 2 μg/mL) was able to protect cells from Cd-derived (20 and 40 μM of CdCl_2_ for 24 h) chromosomal aberrations through its antioxidant property as it protects the cellular environment from free radical damage [149].

- *Curcumin*

Curcumin (IUPAC name: (1E,6E)-1,7-bis(4-hydroxy-3-methoxyphenyl)hepta-1,6-diene-3,5-dione) is a yellow-colored, hydrophobic, polyphenolic compound with the molecular formula C_21_H_20_O_6_ also known as diferuloyl methane. It naturally occurs in turmeric (*Curcuma longa*) and is considered to be a potent anticancer agent. Curcumin was shown to possess powerful radical-scavenging properties and to exhibit protective effects against oxidative damage. Moreover, curcumin has been reported as a potent protective and therapeutic agent against Cd-induced organ toxicity primarily due to its antioxidant properties [150]. Mice exposed to Cd in the dose of either 33 μM/kg or 5 mg/kg b.w. by s.c. injection of single dose CdCl_2_ 1 h or 24 h after the last curcumin treatment, respectively, displayed oxidative stress as the activity of antioxidant enzymes and serum levels of total glutathione and thiol decreased, while levels of MDA and hydrogen peroxide increased. Moreover, Cd exposure led to a decrease in the diameter of seminiferous tubules; however, the lumen diameter of seminiferous tubules increased. Curcumin treatment in the dose of 0.14 mM/kg b.w. for 3 days by gastric gavage or 100 mg/kg b.w. by single i.p. injection, respectively, was performed prior to Cd administration. Mice treated with curcumin were largely protected from the adverse effects of cadmium in terms of lipid and protein peroxidation, the antioxidant defense system was potentiated by curcumin, and morphometrical parameters in the tissues of cadmium-treated mice were partially restored suggesting curcumin as potential therapeutic component against Cd toxicity [151,152].

- *Carvacrol*

Carvacrol (IUPAC name: 2-methyl-5-propan-2-ylphenol) a monoterpenic phenol that is produced by a large number of aromatic plants such as thyme or oregano is considered a safe food additive with various therapeutic applications [153]. Banik et al. [154] investigated the role of carvacrol (100 μM) against Cd-induced apoptosis in PC12 cells. Cell exposure to Cd (10 μM CdCl_2_ for 48 h) led to growth retardation, reduction of glutathione level, and glutathione reductase expression. Moreover, Cd addition resulted in caspase 3 cleavage, enhancement of cytochrome c and apoptosis-inducing factor (AIF), downregulation of expression of the stress regulator kinase mTOR (mammalian target of rapamycin), Akt (protein kinase B), NFKB (nuclear factor kappa-light-chain-enhancer of activated B cells), ERK-1 (extracellular signal-regulated kinase-1), and Nrf2 (nuclear factor erythroid 2-related factor 2). Carvacrol co-exposure strongly reduced cadmium-triggered oxidative stress and caspase-dependent and caspase-independent apoptosis in PC12 cells; hence, carvacrol a natural antioxidant, is suggested as a potential and safe therapeutic compound against the toxicity posed by Cd.

##### Phenolic Acids

Some of the phenolic acids, e.g., ferulic or vanillic acid have been described to mitigate the negative effect of Cd.

- *Ferulic acid*

Ferulic acid (IUPAC name: (E)-3-(4-hydroxy-3-methoxyphenyl)prop-2-enoic acid) a derivative of curcumin is a ubiquitous phenolic compound occurring in cell walls of monocotyledons plants that possess a wide range of therapeutic activities including antiradical potency. The role of ferulic acid (50 mg/kg b.w. orally administered for 15 and 30 days) against Cd-induced oxidative stress in the liver and kidney was investigated in rats exposed to subcutaneously administered Cd (10 mg/kg b.w. of CdCl_2_) for 15 and 30 days. Cd exposure reduced food and water intake leading to decreased body weight and serum total protein contents (TPC) and caused histopathological damage in the liver and kidney. Cd-derived hepatonephrotoxicity was noticeable as the level of marker enzymes such as AST, ALT, ALP, or LDH increased together with AST:ALT ratio, uric acid, urea, urea nitrogen, and creatinine content. Moreover, Cd administration led to hepatic and renal oxidative stress indicated by increased lipid peroxidation (MDA levels), lipid hydroperoxides (LOOH), protein carbonyl content (PCC), total oxidant status (TOS), and oxidative stress index (OSI) in liver and kidney tissues. Antioxidant capacity of the liver and kidney dramatically decreased upon Cd exposure as evidenced by a significant decrease in the levels of total thiols (TTH), total antioxidant concentration (TAC), enzymatic antioxidants (SOD, CAT, and GPx), and non-enzymatic antioxidants (reduced glutathione (GSH) and total free sulfhydryl groups (TSH)). Ferulic acid treatment significantly restored the serum total protein content and renal marker enzymes to normal levels, reduced the oxidative stress markers, and enhanced the levels of antioxidant defense in the liver and kidney. Moreover, ferulic acid alleviated the toxicity of Cd as it protected the normal histological architecture of the liver and kidney tissues. Additionally, Cd-mediated upregulation of TNF-α, COX-2, and HSP70 proteins has been restored by ferulic acid treatment [155].

- *Vanillic acid*

Vanillic acid (IUPAC name: 4-hydroxy-3-methoxybenzoic acid) is an oxidized form of vanillin as it is an intermediate compound in the production of vanillin from ferulic acid. It is a phenolic derivative found in various edible plants and fruits. Its antioxidant, anti-mutagenic, antimicrobial, or anti-inflammation properties have been confirmed by numerous studies [156,157,158]. One crop plant that is particularly hampered by Cd toxicity is rice; hence, Bhuyan et al. (2020) [159] analyzed the effect of vanillic acid on Cd-derived stress in rice (*Oryza sativa* L. cv. BRRI dhan54). Rice seedlings treated with either high (2 mM) or moderate (1 mM) CdCl_2_ concentration for 72 h, showed reduced photosynthetic pigment contents, osmotic status inside the cell, biomass accumulation, and growth. Cadmium treatment changed leaf relative turgidity, ascorbate pool size, increased signs of oxidative stress manifested by enhanced ROS generation and altered antioxidant and glyoxalase systems. Concomitant administration of vanillic acid (50 μM) enhanced antioxidant and glyoxalase enzyme activity, suppressed ROS production, improved osmotic status, increased phytochelatin content, and the biological accumulation factor, and Cd translocation factor by facilitating nutrient homeostasis in rice seedlings. Thus, vanillic acid can be suggested as phytoprotective compound capable to enhance Cd resistance in rice at the early seedling stage.

#### 3.3.2. Hormones, Phytohormones, Metabolites

##### Salicylic Acid

Salicylic acid (IUPAC name: 2-hydroxybenzoic acid) a plant hormone that plays an important role in plant growth and development is a key signal molecule that regulates plant defense responses against pathogens of various origins. Pretreatment with salicylic acid showed a mitigating effect on Cd damage as reviewed by Guo et al. (2019) [160]. Salicylic acid signaling is mainly associated with modulations of reactive oxygen species (ROS) production in plant tissues leading to a series of adaptive responses such as cell wall reconstruction, balancing the uptake of ions including Cd, cheering up the antioxidant defense system, controlling photosynthesis and senescence. However, an excessive dose of exogenous SA might lead to an enhancement of Cd toxicity, thus proper dosing is essential. Li et al. (2019) [161] in their study used 600 μM salicylic acid sprayed on leaves for 10 days to analyze its effect against Cd-derived toxicity caused by 200 μM CdCl_2_ co-exposure in the potato (*Solanum tuberosum* L.) cultivar “Zaodabai” stem explants. Notably, the authors proved that exogenous application of salicylic acid alleviates Cd-induced toxicity as it stimulated the antioxidant enzymatic mechanism pathway, and increased the relative water content (RWC), chlorophyll, proline, and endogenous contents of salicylic acid. Moreover, the content of MDA, hydrogen peroxide (H_2_O_2_), and superoxide anion radicals (O^2·^) decreased upon salicylic acid co-treatment of Cd-treated potato plants. Hence, the salicylic acid application represents a promising treatment in helping plants to defend the Cd toxicity.

##### Abscisic Acid

Abscisic acid (IUPAC name: (2Z,4E)-5-(1-hydroxy-2,6,6-trimethyl-4-oxocyclohex-2-en-1-yl)-3-methylpenta-2,4-dienoic acid) is considered as one of the most important phytohormones that yields abiotic stress tolerance in crop plants. In stress conditions its content in plants increases considerably, mediating stress tolerance function to help plants in stress adaptation, and survival under stressful environment [162]. As plant hormones can play significant role in improvement of plant’s tolerance to abiotic stress, Dawuda et al. (2020) [163] investigated the role of abscisic acid in Cd-induced toxicity in cadmium-sensitive lettuce cultivar (*Lactuca sativa* L., cv. Lüsu). Plants subjected to 100 μM CdCl_2_ (through the hydroponic nutrient solution) displayed markers of oxidative stress as the contents of H_2_O_2_ and MDA increased. Additionally, Cd exposure led to a decrease in photosynthesis, plant biomass, essential nutrients content (except for Cu) in the leaves, while the content of Cd in the leaves and roots of the plants increased. Plant treatment with exogenous abscisic acid (10 μg/L) sprayed on leaves significantly alleviated Cd-induced stress. Abscisic acid co-treatment of Cd-exposed plants increased the activity of antioxidant enzymes, photosynthesis, plant biomass, and amounts of essential nutrient elements, while Cd content in leaves and roots decreased. Presented results suggest that foliar treatment with abscisic acid can enhance Cd tolerance of Cd-sensitive lettuce and thus promote the nutritional quality and food safety under Cd-derived stress.

##### Melatonin

Melatonin (IUPAC name: N-[2-(5-methoxy-1H-indol-3-yl)ethyl]acetamide) a hormonal substance, present in all living organisms including microorganisms, animals, and plants was first discovered in 1958 in the “bovine pineal” gland. It has been demonstrated that melatonin is synthesized not only by the pineal gland, but also by different tissues and organs and, as recently discovered, by mitochondria of every cell. In addition to its predominant circadian rhythm regulatory activities, melatonin has been identified as an antioxidant agent possessing the great potential to protect organisms at very high oxidative stress conditions resulting in cytoprotection, tumor prevention, and immunomodulation [164]. Melatonin-derived protection against abiotic stress has also been described. Cd (25 µM CdCl_2_ for 5 days, or 50 µM Cd(NO_3_)_2_ for 8 days after 2 days of melatonin treatment, respectively), caused toxicity in rapeseed seedlings and mallow (*Malva parviflora*) plants leading to decreased height, biomass, and antioxidant enzyme activity was alleviated by application of 15, 50, or 100 µM of melatonin in the nutrition solution. The antioxidant protection of melatonin from Cd stress is covered by the increase in SOD, CAT, peroxidase, ascorbate peroxidase, proline, chlorophyll, and anthocyanin content, as well as photosynthesis rate, while MDA and H_2_O_2_ levels decreased. Additionally, melatonin enhanced plant tolerance to Cd stress as it reduced organelle and vacuolar fractions of Cd [165,166]. Similarly, melatonin (100 µM) treatment of Malus plants concomitantly exposed to Cd (30 µM CdCl_2_ for up to 20 days) altered the mRNA levels of several genes regulating Cd uptake leading to a reduction in Cd translocation, a decrease in root Cd uptake and leaf Cd accumulation, while root, stem, and leaf melatonin contents increased. Moreover, melatonin application increased concentrations and activities of antioxidant enzymes to mitigate Cd-induced oxidative stress [167]. Consistent with previous findings, melatonin (50 and 200 µM), due to its strong antioxidant capacity, enhanced the capability of the wheat seedling roots to degrade the endogenous hydrogen peroxide overproduced upon Cd (50, 100 µM CdCl_2_) application for 2 days, thus maintained the hydrogen peroxide homeostasis and promoted the primary root growth [168]. Experiments with Chinese cabbage seedlings exposed to Cd (20 µM CdCl_2_ in the nutrient solution for 8 days) revealed that Cd-induced elevation of nitric oxide (NO) that in turn increased the expression of the transporter gene IRT1, consequentially led to the increase in Cd absorption. Melatonin (100 µM sprayed on leaves once a day) co-treatment reduced Cd-triggered NO accumulation and the activity of enzymes related to NO synthesis together with the expression of the IRT1 gene resulting in the decrease in Cd concentration. Moreover, Cd-mediated decrease in photosynthetic parameters and biomass of plants were significantly increased by melatonin [169]. Interestingly, Nabaei and Amooaghaie (2020) [170] exposed *Catharanthus roseus* (L.) G. Don plants to 50, 100, and 200 mg CdSO_4_/kg soil for 30 days and have shown that foliar spray of the NO donor sodium nitroprusside (200 μM) alone or melatonin (100 μM) or their combination enhanced Cd tolerance and phytoremediation efficiency of the exposed plant. The authors concluded that melatonin and/or SNP treatment increased shoot biomass and the content of chlorophyll *a* and chlorophyll *b*, enhanced POX and CAT activities, decreased electrolyte leakage (EL), and optimized the balance of essential cations in leaves through elevation of Cd uptake and translocation from root to shoot. The discrepancies in the NO effect on Cd sequestration might arise from differences in the used Cd concentrations and the experimental setup. It is worth noting that sulfur (S) metabolism plays crucial role in melatonin-mediated Cd tolerance in *Solanum lycopersicum* L. Tomato plants with a silenced caffeic acid O-methyltransferase (COMT) gene resulting in melatonin deficiency under Cd-induced stress (by exposure to 100 µM Cd^2+^ for 15 days 24 h after the first melatonin application in the co-treated plants) displayed decreased S accumulation and elevated Cd phytotoxicity. Application of exogenous melatonin (100 µM by foliar spraying for fifteen days every fifth day) or overexpression of COMT resulted in enhanced uptake and assimilation of S resulting in improved plant growth and Cd tolerance [171].

Melatonin-mediated elevation of Cd tolerance was experimentally studied in mushrooms. Exogenous melatonin (50, 100, or 200 μM) promotes antioxidant activity upon Cd-induced oxidative stress (upon addition of 2, 5, and 8 μM CdCl_2_ for 5 days). Melatonin defense activities directed to decrease Cd toxicity include enhancement of amino acid and glutathione metabolism, oxidation-reduction processes, metal, and ROS detoxification [172].

Studies of Cd toxicity mitigation by melatonin include analyses of animal samples. Mice exposed to Cd (5 mg/kg of CdCl_2_ by everyday i.p. injection) for 14 days displayed ovulation dysfunction and ovarian injury due to increased ER stress. Administration of melatonin (25 mg/kg) has a protective effect on mouse ovaries as it partially reversed Cd-evoked pathohistological damage and increased the number of ovulated oocytes [173]. Strikingly, melatonin (1 μM) is able to inhibit the proliferation of estradiol (E2)-derived proliferation of ovarian cancer cells caused by Cd (1–100 nM of CdCl_2_ for 48 h) suggesting the positive role of melatonin as a preventive compound against Cd-induced ovarian cancer [174].

Cd toxicity to the bone tissue has been analyzed in rat bone (femur) exposed to chronic low-grade Cd (50 mg/L of CdCl_2_ in drinking water for 1 month) and in human adipose (hMSC) cells treated with 0.25 to 50 μmol/L CdCl_2_ for 4 to 72 h depending on the experiment. Cd exposure to adult rats led to decreased mineral and organic components, and reduced Ca^2+^ levels in the femur resulted in bone damage and histological alterations. Melatonin (3 mg/L) treatment ameliorated mineralization and histological structure of the femur. Moreover, Cd addition to growth media of hMSC cells impaired the osteogenic pathway by stimulation of adipogenic differentiation resulting in the premature aging of the bone a clear indicator of toxicity. Melatonin (10 nmol/L to 50 μmol/L) significantly rescued the osteogenic differentiation properties of these cells [175].

Chronic exposure of Cd (1 mg/kg of CdCl_2_) to male and female rats for 8 weeks by intraperitoneal injection was associated with behavioral disorders. Cd administration has anxiogenic-like effects in both anxiety tests and depressive-like effects in the forced swimming test (FST) and leads to memory and learning disabilities in the Y-maze and Morris water maze (MWM) tests. Administration of Cd led to oxidative stress indicated by increased levels of nitric oxide (NO) and lipid peroxidation. Activities of antioxidant enzymes CAT and SOD in the hippocampus were significantly decreased and histopathological studies of the hippocampus showed Cd-mediated neuronal loss in the CA3 sub-region. Melatonin treatment (4 mg/kg 30 min prior to Cd administration) due to its antioxidant properties in the hippocampus counteracted the neurobehavioral disorders and markedly reduced the Cd-induced neuronal loss. Interestingly, the effects of Cd and melatonin are sex-dependent as Cd is more harmful in males, while melatonin is more protective in females [176].

An important impact of Cd exposure to mammals is acute and chronic liver injury and hepatocyte death. Male mice exposed to Cd (2.0 mg/kg of CdCl_2_ by i.p. injection and samples were taken 6, 12, and 24 h after injection) suffered from hepatocellular damage, increased serum ALT/AST enzymes, and inflammatory cell death mediated by the NOD-like receptor pyrin domain containing 3 (NLRP3) inflammasome. Melatonin (10 mg/kg by i.p. injection for 3 days before Cd administration) treatment via its antioxidant activity decreased serum ALT/AST levels leading to alleviation of Cd-induced liver injury, suppressed pro-inflammatory cytokine production, inhibited NLRP3 inflammasome activation, and attenuated hepatocyte death. Great attention has to be paid to melatonin-mediated abrogation of Cd-induced overexpression of the key endogenous regulator of the cellular redox balance, thioredoxin-interacting protein (TXNIP) as it plays an important role in the pathogenesis of acute liver failure. Moreover, melatonin decreased the interaction between TXNIP and NLRP3. It can be therefore concluded that melatonin protection against Cd-triggered liver inflammation and hepatocyte death is mediated through inhibition of the TXNIP-NLRP3 inflammasome pathway [177].

##### Fulvic Acid

Fulvic acid (IUPAC name: 3,7,8-trihydroxy-3-methyl-10-oxo-1,4-dihydropyrano [4,3-b]chromene-9-carboxylic acid) is a subclass of diverse compounds known as humic substances, products of decomposition and formed through geochemical and biological reactions, such as the breakdown of biological waste in a compost heap. Fulvic acid was shown to play important role in immune system regulation, oxidative state maintenance, and improvement of gastrointestinal function, and, in addition, fulvic acid possesses a strong complexation ability with heavy metals [178,179,180]. Hence, Wang et al. (2019) [181] analyzed the potential protective effect of fulvic acid on Cd toxicity in lettuce seedlings. Seedlings in the three-leaf stage were treated with 20 μM CdCl_2_ in hydroponics for 2 weeks which caused growth inhibition due to nutrient elemental imbalance, reduction of the photosynthetic pigment resulting in the disorganization of the photosynthesis apparatus, oxidative stress caused by the accumulation of ROS. Application of 0.5 g/L fulvic acid to Cd exposed plants resulted in stimulation of the antioxidant capacity that in turn inhibited ROS accumulation. Additionally, fulvic acid treatment enhanced photosynthetic activity by protecting PSII against Cd toxicity, accelerating chlorophyll biosynthesis, and promoting translocation of the elemental nutrients involved in photosynthesis, such as Fe, Zn, and Mn from roots to shoots. Thus, the authors assume that the ability of fulvic acid to mitigate Cd-triggered toxicity in lettuce is due to the elimination of ROS overproduction and suppression of Cd uptake.

The toxic effect of Cd on the tested systems and its alleviation by bioactive substances supplementation is summarized in Table 3.

### 3.4. Whole Plant Extracts

**Figure 2:**
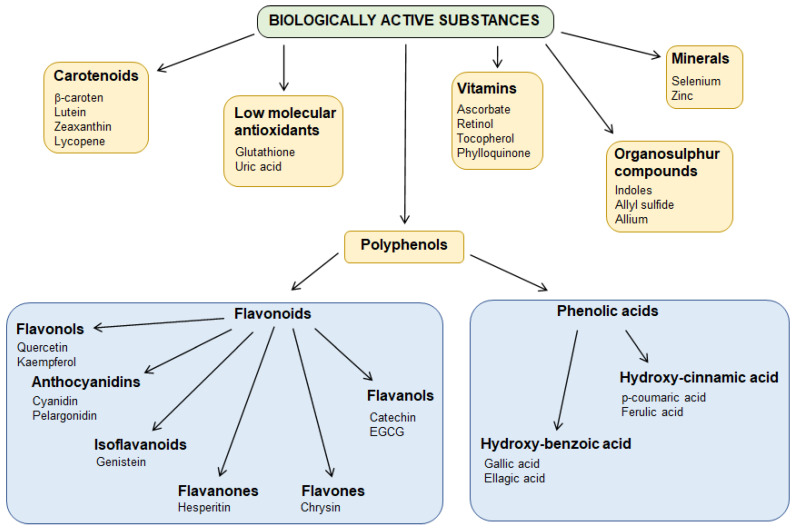
Bioactive substances. Schematic draw of the most abundant bioactive substances in whole plant extracts.

As plenty of plant species possess strong antioxidant activities (Figure 2), the possible effect of various whole plant extracts on Cd toxicity elimination has been investigated.

#### 3.4.1. *Senna alexandrina* Extract

*Senna alexandrina* belongs to the Caesalpiniaceae family, it is a small, perennial plant that grows in North Africa. As the analysis of *Senna alexandrina* leaves demonstrated the presence of several pharmacologically active molecules including apigenin-6,8-di-C-glycoside, tinnevellin glycoside, emodin-8-O-beta-D-glucopyranoside, aloe emodin, isorhamnetin-3-O-beta-gentiobioside, kaempferol, and D-3-O-methylinositol it is mainly used as antibacterial, antiviral, or laxative substance. In the study of Wang et al. (2020) [182] authors used *S. alexandrina* extracts (SAE) prepared from finely powdered plants soaked in 70% methanol, subsequently concentrated and lyophilized, to investigate the effect of SAE against Cd-induced hepatotoxicity in rats. Rats exposed to Cd (0.6 mg/kg/day of CdCl_2_ intraperitoneally injected for 28 days) displayed significantly increased serological AST, ALT, and TB levels, also levels of oxidative markers (LPO, MDA, NO), pro-inflammatory cytokines (IL-1β and TNF-α), and pro-apoptotic molecules (BAX and caspase-3) increased, while the activity of antioxidant molecules (GSH, GPx, GR, SOD, and CAT) and anti-apoptotic protein Bcl-2, decreased. SAE (100 mg/kg administered orally for 28 days) supplementation upregulated expression of the most prominent cellular antioxidant regulatory molecule Nrf2, precluded Cd deposition to the liver, and recovered Cd-mediated oxidative, inflammatory, and apoptotic responses of the organism. Moreover, SAE treatment prevented histopathological changes in the liver tissue caused by Cd. Results of the study indicate that SAE via enhancement of Nrf2 expression triggers initiation of anti-inflammatory and anti-apoptotic cascades and thus protects liver tissue against Cd toxicity.

#### 3.4.2. *Portulaca oleracea* (Purslane) Extract

*Portulaca oleracea* a member of the *Portulacaceae* family is a medicinal plant known to be rich in omega-3 fatty acids such as α-linolenic acid and eicosapentaenoic acid, and alkaloid pigment betalain which shows antioxidant and antimutagenic properties. Moreover, it contains carotenoids, vitamins A, C, and B, and mineral elements including Mg, Ca, K, and Fe. Ethanolic extract prepared from dried and powdered leaves of purslane soaked in 50% ethanol was used by Seif et al. (2019) [183] to investigate its effect on Cd-induced hepato-nephrotoxicity in rats. Concomitant with others, rats exposed to Cd (3.5 mg/kg/day b.w. of CdCl_2_ intraperitoneally injected for 30 days) suffered from oxidative stress as Cd triggered reduction in antioxidant enzymes (SOD, CAT, GPX) activities, while lipid peroxidation increased, as indicated by the increase in MDA content in the serum. Histological analyses of the liver and kidney revealed that Cd-mediated bleeding and congestion resulted in serious hepatic and renal tissue necrosis and degeneration. Additionally, the evident hepatocellular damage was manifested by marked enhancement of serum AST, ALT, ALP, TGi activities, and TP levels. Alteration of the kidney function by Cd exposure eventuated in serum urea and creatinine elevation. Purslane extract (PEE) supplementation (2 g/kg b.w.) triggered either complete restoration or large improvement of all determined parameters and tissues altered by Cd. Thus, it can be concluded that PEE provides substantial protection against Cd-mediated hepato-nephrotoxicity in the animal model system.

#### 3.4.3. *Aronia melanocarpa* L. Extract

Berries of *Aronia melanocarpa* L. (chokeberries) a perennial shrub of the *Rosaceae* family represent a rich source of powerful natural antioxidants: polyphenols. Considering the large number of hydroxyl groups, these molecules are designed to chelate metal ions such as Cd. In addition to polyphenols, chokeberries are abundant in other bioelements including vitamins, triterpenes, carotenoids, and phytosterols capable to detoxify free radicals and ROS. The beneficial impact of the *A. melanocarpa* extract (AE) (as 0.1% aqueous solution in drinking water) against Cd-induced toxicity (1 or 5 mg/kg b.w. of CdCl_2_ in the diet for 3, 10, 17, and 24 months) in rats was investigated in the liver by Mężyńska et al. (2019) [184] with the major focus on the antioxidant capacity of the liver, and Kozłowska et al. (2020) [185] focused on the amount of collagen type I and III in the liver, submandibular gland by Dąbrowski et al. (2020) [186], mineral element metabolism by Borowska et al. (2017) [187]. AE significantly protected model animals from Cd-derived oxidative stress as it improved the enzymatic and nonenzymatic antioxidant apparatus, increased total antioxidant status, and reduced lipid peroxidation and Cd accumulation in the liver tissue and submandibular gland. Collagen metabolism affected by Cd exposure was significantly improved by AE supplementation resulting in the correction of collagen types I and III expression and their mutual ratio (collagen III/collagen I); moreover, AE treatment increased concentrations of MMPs (matrix metalloproteinases) and their TIMPs (tissue inhibitors) in the liver. In addition, the metabolism of biologically important elements such as Zn or Cu impaired by Cd was significantly (completely or partially) improved by AE supplementation. The results of the studies suggest that the consumption of *Aronia* products may offer considerable protection against multiple injuries triggered by Cd exposure.

#### 3.4.4. *Physalis peruviana* L. Extract

Fruits of *Physalis peruviana*, also known as goldenberry, possess a noticeable antioxidant property due to their rich content of polyphenols, vitamins C, E, A, D, and some of the B vitamins, withanolides, and carotenoids.

The effect of *Physalis peruviana* methanolic extract (methanol was removed under reduced pressure) against nephrotoxicity and hepatotoxicity caused by Cd in rats was investigated by Dkhil et al. (2014) [188].

Methanolic extract (MEPh) was pre-administered in a dose of 200 mg/kg b.w. to a group of rats exposed to intraperitoneal injection of 6.5 mg/kg/day CdCl_2_ for 5 days. Cd-mediated significant decrease in kidney weight and index, an increase in liver enzymes, and the levels of bilirubin, uric acid, urea, and creatinine were corrected by MEPh supplementation. Hence, MEPH-triggered antioxidant and anti-apoptotic effects alleviated hepatorenal toxicity in Cd-treated rats. This indicates that fruits of *Physalis peruviana* may provide suitable protection against Cd toxicity.

#### 3.4.5. *Mimosa caesalpiniifolia* Extract

*Mimosa caesalpiniifolia* (Mimosa) is a perennial fast-growing plant native to northeastern Brazil typical for its high regeneration capacity and drought resistance. It belongs to Mimosoid legumes that comprise more than 500 species. Interestingly, apart from being used in traditional medicine, it provides a source of nourishment for cattle mainly during the dry season, or it is used as a material for strong and resistant wooden constructions. Silva et al. (2014) [189] investigated the predicted positive role of mimosa extract and ethyl acetate fraction toward Cd-mediated stress in rats. Rats exposed to Cd (1.2 mg/kg b.w. CdCl_2_ for 15 days) suffered from DNA damage in peripheral blood cells and liver cells due to elevated oxidative stress. Alcoholic extracts of mimosa at 62.5 and 125 mg/kg/day similarly to ethyl acetate extract (62.5 mg/kg/day) significantly reduced Cd-mediated genotoxicity suggesting its protective effect on DNA integrity maintenance under Cd contamination.

#### 3.4.6. *Spirulina platensis* Extract

The single-celled alga *Spirulina platensis* which is characterized by strong antioxidant and anti-inflammatory properties grows in freshwater and serves as a source of food. *S. platensis* has been shown to have great therapeutic value as it possesses hepatoprotective, and anti-mutagenic properties, and due to its high iron and vitamin contents to reduce anemia. Moreover, it has the ability to reduce heavy metal toxicity and detoxify nephrotoxic compounds in the body. Aly et al. (2018) [190] used *S. platensis* extract to investigate its effect on Cd-induced chromosome aberrations and DNA injury in rats. The toxic effect of Cd (3.5 mg/kg b.w. CdCl_2_ dissolved in 2 mL distilled water, for 60 days) has been demonstrated by the ISSR-PCR analysis that revealed various genetic impairments such as marked DNA fragmentation, deletion, disappearance, or appearance of some DNA base pairs. Furthermore, Cd addition induced the reduction in chromosome number, chromosomal fragmentation, and ring formation, deletions of chromatid, and generation of dicentric chromosomes. Strikingly, the bone marrow and ISSR-PCR analysis of rats treated with *S. platensis* extract (1 g/kg dissolved in 5 mL distilled water) showed significant reduction of Cd-mediated genetic defects represented by the decrease in the number of chromosomal aberrations and overall DNA stabilization. Results of the study indicate that S. platensis extract protects rats against Cd-derived genotoxicity.

#### 3.4.7. Purple Carrot Extract

Carrot (*Daucus carota* L.) cultivars belong worldwide to one of the most economically important crops relevant not only for human but also for animal nutrition. Several taproot types differing in the color can be recognized. Different taproot color is commonly associated with different crop composition. Red carrots contain lycopene, while yellow carrots contain a high amount of lutein, orange carrots are rich in α-and β-carotene, whereas polyphenols, predominantly anthocyanins are typical for the purple color of the carrot. Claudio et al. (2016) [191] exposed rats to Cd by a single intraperitoneal injection of 1.2 mg/kg b.w. CdCl_2_ and analyzed after 15 days if the concomitantly injected extract of purple carrot in the concentration of 400 and 800 mg/L reduces Cd-mediated alterations in multiple organs of the exposed animals. Histopathological sections unveiled liver tissue degeneration upon Cd addition which was significantly improved by the purple carrot extract treatment. Similarly, a positive effect of the extract was observed by comet and micronucleus assay as it showed elimination of the Cd-induced genetic alterations in blood and hepatocytes. Moreover, the Cd-mediated decrease in the CuZn-SOD and cytochrome C gene expression and increase in the 8OHdG levels in liver cells were improved upon purple carrot extract treatment. This suggests that extract of the purple carrot has the ability to protect multiple rat organs from genotoxicity, oxidative stress, and tissue degeneration triggered by Cd.

#### 3.4.8. Grape Seed Extract and Grape Seed Proanthocyanidins

Grape (*Vitis vinifera* L.) consumed by humans as fruit or processed as wine is a member of *Vitaceae*. Seeds of grape used for the production of grape seed extract (GSE) are rich in natural polyphenolic compounds containing dimers, trimers, and other oligomers of catechin and epicatechin, gallocatechin, and epigallocatechin. These substances are types of proanthocyanidins or condensed tannins that belong to flavanols or flavan-3-ols. Importantly, proanthocyanidins belong to considerable pharmacological substances as they dispose of antioxidant, antibacterial, antiviral, antitumor, or antithrombotic activities, in addition to anti-inflammatory, and antitoxic effects. Moreover, it has been reported that these compounds have the ability to battle against atherosclerosis, large bowel cancer, gastric ulcer, cataracts, diabetes, and modulate prostatic oxidative stress. Grape seed proanthocyanidins (GSP) belong to one of the most efficient antioxidants as they defend cells from oxidative damage by controlling the antioxidant status maintenance and elimination of the pro-inflammatory mediator transfer. Strikingly, GSP possesses stronger antioxidant properties compared to vitamin C, vitamin E, and β-carotene. Several studies have been performed to analyze the effect of GSE and GSP on Cd toxicity. Different aspects of Cd-induced toxicity on rat testes and the impact of GSE or GSP treatment have been investigated by [192,193,194,195]. Rats exposed to Cd (2.5 or 5 mg/kg b.w. of CdCl_2_ for 4 weeks or 3 months, respectively), displayed markers of oxidative stress due to decreased expression of the cytoprotective molecules Nrf2/HO-1 through the PI3K/Akt-dependent pathway leading to testicular dysfunction. This was manifested by the marked reduction in the mRNA expression levels of steroidogenesis-associated genes such as cytochrome P450 cholesterol side-chain cleavage enzyme, cytochrome P450 17A1, 3β-hydroxysteroid dehydrogenase (3β-HSD), 17β-HSD, androgen receptor, steroidogenic acute regulatory protein, and follicle-stimulating hormone receptor [190,192]. Furthermore, rat exposure to CdCl_2_ (2.5 or 5 mg/kg b.w. for 48 h or 30 days, respectively), led to the elevation of the number of apoptotic cells and testicular histoarchitecture alteration represented by the decrease in the mean seminiferous tubules diameter and irregular arrangement of their epithelial lining, testes congestion, edema, degeneration of the spermatogenic cells resulting in the impaired spermatogenesis [192,194]. Importantly, GSE and GSP (100–400 mg/kg b.w.) treatment normalized Cd-induced impairments of rat testes suggesting its protective role against Cd-mediated testicular toxicity.

Other studies investigating Cd’s impact on rats showed its negative effect on the brain, pancreas, and prostate. Cerebrum degeneration upon 3-month administration of 15 mg/kg b.w. CdCl_2_ in drinking water was demonstrated through histopathological analyses showing fibrosis and vacuolations of brain vessels. Moreover, capsular cells underwent satellitosis, levels of MDA in the brain cells increased, while MAO-A, acetylcholinesterase, and GR decreased, and expression of bcl-associated X (Bax) protein, an apoptosis indicator, dramatically increased [196]. Bashir et al. (2016) [197] referred to Cd-induced (5 mg/kg/day b.w. for 4 weeks) toxicity through elevated oxidative stress toward the pancreas. The pancreas of Cd-intoxicated rats displayed signs of apoptosis as the levels of apoptotic markers (cleaved Caspase-12/9/8/3) increased, suffered from oxidative stress indicated by the decreased levels of cellular protective molecules Nrf-2 and HO-1, and enhanced levels of proinflammatory cytokines, including TNF-α, IL1β, and IFN-γ. Histopathological micrographs showed irregularly shaped islets invading the exocrine acini, with most islet cells, especially in the center of the islet, displaying cytoplasmic alterations. Similarly, the prostate of Cd-exposed rats showed marked functional and structural impairments. As shown by Lei et al. (2017) [198] altered levels of MDA, NO, reduced/oxidized glutathione, and enzymatic antioxidant capacity in the prostate of animals administered to CdCl_2_ (60 mg/L in drinking water for 20 weeks) displayed its enhanced oxidative stress. Moreover, Cd addition led to the elevation of transforming growth factor-β1 (TGF-β1) levels, phosphorylation of Smad3 (p-Smad3) in the prostate, a decrease in Smad7, Nrf-2, HO-1, γ-GCLC, and miR-133a/b levels, and caused prostate fibrosis. Rat treatment with GSE or GSP (100–400 mg/kg b.w.) in all experimental conditions attenuated Cd-triggered alterations as it improved the antioxidant capacity of the organism and normalized the histological outlines of the analyzed tissues. Additionally, experiments with mice showed that exposure of 5 mg/kg b.w. CdCl_2_ for 30 days in mice causes inhibition of SOD and GSH-Px activities and increases MDA content in the kidney resulting in renal dysfunction and elevating levels of Bax and Bcl-2 expression suggesting renal cell apoptosis [199]. Interestingly, Cd-mediated oxidative stress altered meiosis progression during oogenesis in chicken as indicated by the impaired expression of meiosis-specific markers such as Stra8, Spo11, Scp3, and Dmc1 at embryonic day E17.5 upon 3 μg CdCl_2_ injection to eggs at E6.5 of fertilized Hyline chicken [200]. Importantly, GSP (50 mg/kg or 150 μg, respectively), treatment mitigated Cd-triggered renal oxidative damage and reproductive disorders. Presented results demonstrate the eminent protective effect of GSE and GSP against Cd-mediated toxicity.

## 4. Conclusions

Cadmium exposure to living organisms is associated with various adverse effects depending on the dose, time of exposure, and the model system. Plants affected by Cd display growth retardation, modified photosynthetic capacity, oxidative stress resulting from enhanced ROS generation and altered function of the enzymatic and non-enzymatic antioxidant system. Moreover, Cd-derived homeostasis destruction was noticed in a single-celled microorganism, or cell lines. Cells exposed to Cd suffered from ruined ionome balance and oxidative stress that resulted in membrane lipid peroxidation causing cell shape alterations leading to growth impairments. Aquatic and terrestrial animals exhibit negative affection of Cd predominantly from contaminated environment. Aquatic animals show more pronounced danger in Cd accumulation from the contaminated water resulting in blood, kidney, or liver toxicity as Cd-mediated oxidative stress leads to organ damage and immunotoxicity. The major disorders uprising from Cd exposure in the terrestrial animal model systems belong to tissue toxicity and damage due to Cd-mediated stress causing hepatotoxicity, neurotoxicity, reproductive system, kidney, bone, and cardiac toxicity.

The majority of Cd-derived alterations are associated with the destruction of the antioxidant defense system, in turn leading to oxidative stress. Therefore, mitigation strategies to eliminate the impact of Cd on the organism need to be applied. One of the most promising strategies represents the food supply system as the biologically active substances abundant in health-promoting foods, in particular, the antioxidative nutrients are responsible for the elimination of Cd toxicity.

The findings summarized above represent only a small portion of the studies that have described the preservation of the organism, particular organelle, or cellular function altered by Cd exposure with the use of bioactive substances primarily possessing antioxidant properties and/or metal-chelating capability. The vitamins with antioxidant properties (such as C and E), trace elements (including Zn, Mg, Se, Mn, or Ca), certain naturally occurring compounds and phytochemicals efficiently prevent or mitigate the cytotoxic effects of Cd although the mitigation effect might be limited by the permitted dose of the bioactive substance. Therefore, we can assume that as any single molecule will not completely inhibit Cd toxicity, the administration of a mixture including several bioactive substances may serve as a more effective strategy to counter Cd toxicity.

## Figures and Tables

**Table 1:** Effects of vitamins.

Substances	Model	Dose of Substances	Dose of Cd	Detrimental Response to Cd Exposure and the Curative Effect of the Substance	Ref.
Vitamin C	*Zea mays* L.	0.1–0.5 mM (4 weeks foliar application)	50 mg kg^−1^ soil	Plant growth, photosynthesis, protein content, MDA content, H_2_O_2_ accumulation, CAT, SOD, GR activities, grains Cd uptake	Zhang et al., 2019 [51]
	*Brassica napus* L.	250 and 500 mg kg^−1^ (single foliar application)	10 μM (equal dose)	Ascorbate—glutathione cycle, ROS content	Jung et al., 2020 [53]
	*Triticum aestivum* L.	1 mM (seedlings preincubation)	100 μM (equal dose)	AsA-GSH-NADPH cycle homeostasis, ROS generation	Wang et al., 2017 [52]
	*Schizosaccharomyces pombe* L.	10 mM (cell treatment for 30 min)	10–400 μM (equal dose)	Ionome balance, CAT and SOD activity, MDA content, cell morphology	Navratilova et al., 2021 [54]
	Rabbit	150 mg kg^−1^ b.w. (oral administration for 28 days)	1.5 mg kg^−1^ b.w. (oral administration for 28 days)	Cd accumulation, blood biochemical parameters	Mumtaz et al., 2019 [55]; Ali et al., 2020 [56]
	Mouse	50 and 100 mg kg^−1^ b.w.	100 mg L^−1^ (in drinking water for 8 weeks)	Vascular disfunction, hypertension, oxidative damage	Donpunha et al., 2011 [57]
Vitamin E	Tilapia	50 mg kg^−1^ diet for 12 weeks	20 and 50 mg kg^−1^ diet	Serum creatine, ALT and AST activity, residual Cd content	Ayyat et al., 2017 [61]
	*Ctenopharyngodon idellus*	20 IU kg^−1^ (single dose at day 4 after Cd injection)	20 μM kg^−1^ single i.p. injection	Hepatocytes effect, MDA content, hepatocytes apoptosis and apoptosis-related gene, liver morphology, SOD, CAT, GPx activity	Duan et al., 2018 [62]; Huang et al., 2020 [63]
	Mice	100 mg kg^−1^ or 50 IU kg^−1^	5 mg kg^−1^ b.w. for 28 days, or 30 ppm for 7 weeks in drinking water	Liver weight and Cd content, ALT and AST content, hepatocellular necrosis, rupture in hepatocytes, inhibition of oxidative stress, antioxidant enzymes and expression of Nrf2 genes	Fang et al., 2021 [64]; Al-Attar 2011 [65]
	Rat	20 mg kg^−1^ b.w., 100 mg kg^−1^ b.w., 250 mg kg^−1^ b.w., or 40 and 400 mg kg^−1^ b.w., and 100 UI kg^−1^)	20 mg kg^−1^ b.w. for 30 days, and 5 mg kg^−1^ b.w. for 28 days in the daily gavage; or i.p. 2 mg kg^−1^ b.w. for 8 or 28 days, or in drinking water 50 ppm for 20 weeks)	CAT, SOD, GR, GPx and GST, LPX, body weight effect, serum urea and creatinine, renal histological alterations, angiotensin converting enzyme, blood pressure, heart rate	Adi et al., 2016 [66], Fang et al., 2021 [67], Karabulut-Bulan et al., 2008 [68], Choi and Rhee 2003 [69]

**Table 2:** Effects of mineral elements.

Substances	Model	Dose of Substances	Dose of Cd	Detrimental Response to Cd Exposure and the Curative Effect of the Substance	Ref.
Selenium	Chicken	2 mg kg^−1^ or 0.5 mg kg^−1^ Se in the diet	150 mg kg^−1^ for 90 or 120 days	Ionome alterations, amino acid content profile, MDA, DNA and protein crosslink, protein carbonyl content, CYP450, b5, GSH, AND, ERND, AH, cytochrome C reductase, CAT, SOD, GPX, caspase8, MAPK signaling pathway	Qu et al., 2020 [79], Cong et al., 2019 [81], Wang et al., 2020 [82]
	Avian leghorn male hepatoma cells	1.25 or 2.5 μM	2.5 μM (equal dose) for 24 h	Ca^2+^ homeostasis, calmodulin, alteration of the cadherin/calreticulin cycle, ER stress, autophagy leading to cell death	Zhang et al., 2020 [80]
	porcine jejunal epithelial cells	0.4 ppm in the cell culture medium for 24 h	0.5–1 ppm in the media for 24 h	Cell viability, DNA damage, cell death	Lynch et al., 2017 [83]
	*Festuca pratensis* L.	0.1 mg L^−1^ dissolved in water	30 mg L^−1^ for 7 days	Plant height, root length, MDA content, electrolyte leakage, antioxidant enzyme activities, photosynthetic efficiency, chlorophyll, and soluble protein content	Li et al., 2020 [84]
	*Raphanus sativus* L.	2–8 mg L^−1^ for 30 days	5–10 mg L^−1^ for 30 days	Cd uptake, chlorophyll biosynthesis, micronutrients content, enzymatic antioxidant protective system	Auobi Amirabad et al., 2020 [85]
	*Triticum aestivum* L.	10–40 mg L^−1^ (foliar application for 6 months)	0.3 mg kg^−1^ soil	Photosynthesis, plant biomass production, antioxidant enzymes activity	Wu et al., 2020 [86]
Zinc	SH-SY5Y catecholaminergic neuroblastoma cells	50 μM (equal dose for 24 h)	10 μM (equal dose for 24 h)	Attenuate the Cd-induced neurotoxicity	Branca et al., 2018 [92]
	MDBK epithelial cells	10 and 50 μM (equal dose for 24 h)	10 and 50 μM (equal dose for 24 h)	Cd uptake, Cd-mediated apoptotic cell death, mitochondrial damage and oxidative stress	Zhang et al., 2014 [93]
	*Saccharomyces cerevisiae*	40–640 μM (equal dose for 2 h)	40–320 μM (equal dose for 2 h)	Genes expression alternation, ionome homeostasis and mitochondrial membrane potential, sulfur and GSH metabolism, ribosomal proteins, S-containing amino acids, S-rich proteins and antioxidant enzymes	Pan et al., 2017 [94]
	Rats	60 mg L^−1^ in drinking water during gestation and lactation	50 mg L^−1^ in drinking water	Hippocampal volume, CA1, CA3 pyramidal cell layer and the dentate gyrus, SOD, metallothionein level, prenatal Zn metabolism	Mimouna et al., 2018 [95], Chemek et al., 2016 [98]
	*Sinopotamon henanense*	0.1–1 mg L^−1^ for 14 days	0.05–0.5 mg L^−1^ for 14 days	Sperm count and motility, histological damage, morphological lesions, relative testis weight, SOD, CAT and GPx activity, MDA	Liu et al., 2020 [96], Babaknejad et al., 2018 [97]
	*Spodoptera exigua*	100–400 μg g^−1^ of food for 135 generations	44 μg g^−1^ of food for 135 generations	DNA damage, ADP/ATP ratio, ATP and HSP70 concentrations, growth rate	Tarnawska et al., 2019 [99]
	*Triticum aestivum* L.	0.2% foliar spray, or 41.2 mg kg^−1^ soil	naturally contaminated water	Influx transporter gene TaNramp5, efflux transporters TaTM20 and TaHMA3, leaf TaHMA2, root TaLCT1 gene	Zhou et al., 2020 [101]
	*Spinaciae oleracea*	100 mg L^−1^ water for 2 weeks	1 mg L^−1^ water for 2 weeks	Cu and Fe content	Sharifan et al., 2020 [102]
	*Petroselinum sativum*	100 mg L^−1^ water for 2 weeks	1 mg L^−1^ water for 2 weeks	Cu and Fe content	Sharifan et al., 2020 [102]
	*Coriandrum sativum* L.	100 mg L^−1^ water for 2 weeks	1 mg L^−1^ water for 2 weeks	Cu and Fe content	Sharifan et al., 2020 [102]
	*Matricaria chamomilla* L.	0.1–5 mM in hydroponic solution for 2 weeks	120 and 180 μM in hydroponic solution for 2 weeks	ROS accumulation, MDA, antioxidant enzyme, reduced influx of Cd, biomass accumulation	Farzadfar et al., 2013 [105]
	*Arabidopsis thaliana* L.	3 mM (seedlings exposure for 5 days)	50 μM (seedlings exposure for 5 days)	Oxidative stress, cell H_2_O_2_ content, lipid peroxidation, auxin content and distribution, root length	Li et al., 2016 [106]
	*Oryza sativa* L.	2.5 and 5 mg kg^−1^ soil (3 days), and 0.05–5 mM in BMS solution (till ripening stage)	50 and 100 mg kg^−1^ soil (3 days), and 50 μM in BMS solution (till ripening stage)	Cd intake, oxidative stress, SOD, CAT and POD activity, photosynthesis, carotenoids, proline and protein content, grain yield and yield components, *OsNRAMP1* and *OsNRAMP5*, *OsHMA2*, *OsHMA3* transporter genes	Zhang et al., 2020 [107], Kanu et al., 2019 [108]
Calcium	*Cicer arietinum* L.	100 mM (seeds exposure for 6 days)	200 μM (seeds exposure for 6 days)	Oxidative stress, thioredoxin and thioredoxin reductase activities, SOD, CAT and APX	Sakouhi et al., 2021 [109]
	*Hypogymnia physodes* (L.) Nyl.	100 and 1000 μM in water for 24 h	10 and 100 μM in water for 24 h	ROS and MDA, thiol, glutathione, and ascorbic acid levels	Kováčik et al., 2020 [110]
	*Saccharomyces cerevisiae* L.	10 mM (in culture media for 60 min or 2–3 days)	50–500 μM (in culture media for 60 min or 2–3 days)	Ca release from vacuole, Yvc1p transporter, Cch1p/Mid1p channel	Ruta et al., 2014 [111]
	*Bacillus* sp. 98	5–30 mM (in culture media)	0.2–4 mM (in culture media)	Intracellular NO production, NO synthase, NO dioxygenase, Fe uptake	Wu et al., 2021 [112]
	*Oncorhynchus mykiss*	30 and 60 mg g^−1^ food, or 20–60 mg kg^−1^ in diet	50 μg L^−1^ in water for 7 days, or 3 μg L^−1^ in water, or 500 mg kg^−1^ diet for 28 days	Cd uptake, Cd transport, morphology	Baldisserotto et al., 2004 [113], Franklin et al., 2005 [114]
	*Oreochromis mossambicus*	0.2–0.8 mM freshwater concentration	10 μg L^−1^ freshwater, or 10 μg per fish for 35 days	Cd transport, Cd accumulation in gill and gut, morphology	Pratap and Wendelaar Bonga 2007 [115]
	Rat	1–6% in milk or 0.4% solution or 100 mg kg^−1^ orally administered	0.5 mg kg^−1^ b.w. for 10 days 1–50 mg kg^−1^ diet for 90 days or 44 mg L^−1^ in drinking water for 4 weeks	Cd content, ionome, bone formation, expression of osteogenic gene markers, fibroblast growth factor 23/Klotho-associated gene expression Cd-induced hepatotoxicity, MDA, H_2_O_2_, protein carbonyls, interleukin (IL) 1β, IL-6, IL17A, tumor necrosis factor-α, anti-inflammatory IL-10, IL-22 markers, GSH, GPx, CAT activities	Sarić et al., 2002 [116], Huang et al., 2019 [117], El-Boshy et al., 2020 [118]
	Mice	100 mg kg^−1^ diet	10–1000 ppm in the diet for 28 days	Cd-induced nephrotoxicity, glomerular atrophy, renal proximal tubule injury, MDA, urine protein, KIM-1, apoptosi	Gu et al., 2020 [119]
Silicon	*Boehmeria nivea* (L.) Gaud	1 mM in hydrophonic solution for 7 days	5 mg L^−1^ in hydrophonic solution for 7 days	Cd translocation, SOD, CAT, POD, APX, ROS, MDA, H_2_O_2_, glutathione, ascorbate, vitamin E	Tang et al., 2015 [120]
	*Oryza sativa* L.	5–20 mg L^−1^ foliar application	0.84 mg kg^−1^ soil	Cd content, phytic acid	Hussain et al., 2020 [121]
Manganese	*Phytolacca acinosa* Roxb	0.5–12 mM in hydrophonic solution for 17 days	50–200 μM in hydrophonic solution for 17 days	Mn/Cd ratio, lipid peroxidation and plant water-loss, photosynthesis	Liu et al., 2013 [122]
	Mice	20 mg kg^−1^ b.w. single i.p. injection	7 mg kg^−1^ b.w. single s.c. injection	Cd content, GSH-Px activity, lipid peroxidation, GSH, CAT	Eybl and Kotyzová 2010 [123]
Magnesium	Rat blood plasma	3 and 50 mg kg^−1^ b.w. i.p. or oral exposure 10 min or 1 h prior to Cd exposure	30 mg kg^−1^ b.w. single dose administered by orogastric tube or 1.5 mg kg^−1^ b.w. i.p. injection of single dose	SOD activity, superoxide anion, total oxidative status, MDA and oxidation protein content	Buha et al., 2012 [125]
	Rat	0.5 or 1.5 mg kg^−1^ b.w. i.p. injection for 21 days	1 mg kg^−1^ b.w. i.p. injection for 21 days	Cd-induced nephrotoxicity, MDA, serum sodium, potassium, and urea levels, creatinine, and protein levels	Babaknejad et al., 2016 [126]
	Isolated perfused rat liver model system	1.2 mM for 90 min	15 μM for 90 min	Glutathione level, enhanced MDA content and aminotransferase activity	Ghaffarian-Bahraman et al., 2014 [127]

**Table 3:** Effect of bioactive substances.

Substances	Model	Dose of Substances	Dose of Cd	Detrimental Response to Cd Exposure and the Curative Effect of the Substance	Ref.
Rutin	Rat	25–100 mg kg^−1^ b.w. (oral administration for 14 or 30 days)	5 mg kg^−1^ b.w. (oral administration for 14 or 30 days)	Neurotoxicity and cognitive disturbance, ERK1/2 and JNK apoptotic pathways, PTEN derived regulation of mTOR survival pathway, ectonucleotidases, adenosine deaminase and MAO activities	Oboh et al., 2019 [130], Abdel-Aleem and Khaleel 2018 [131]
Chrysin	Mice	2.5 and 5 mg kg^−1^ b.w. in drinking water for 30 days	2 mg kg^−1^ b.w. in drinking water for 30 days	Hepatic stress, liver enzymes enhancement, morphological changes	Beyrami et al., 2020 [132]
Diosmin	Rat	100 mg kg^−1^ b.w. in drinking water for 30 days	200 ppm in drinking water for 30 days	Hepatotoxicity, liver enzyme, antioxidant parameters, histopathological parameters	Ağır and Eraslan 2019 [133]
Quercetin	Mice	5–100 mg kg^−1^ b.w. by i.p. injection for 3 days	0.4 mg kg^−1^ b.w. by i.p. injection for 3 days	Autophagosome formation, LC3-II/β-actin ratio, ROS, MDA content, antioxidant capacity	Yuan et al., 2016 [134]
	Primary rat proximal tubular cells	1 μg mL^−1^ for 12 h	2.5 μM for 12 h	Autophagy marker proteins, TFEB-dependent recovery of lysosomal function, v-ATPases	Zhao et al., 2021 [136]
	Rat	10 and 50 mg kg^−1^ b.w. by oral gavage for 12 weeks 50 mg kg^−1^ b.w. i.p. injection for 4 weeks	4.89 mg kg^−1^ b.w. in drinking water for 12 weeks 2 mg kg^−1^ b.w. i.p. injection for 4 weeks	Nephrotoxicity, metabolic alterations, lipids, amino acids, and purine metabolism Decreased body and testicular weight, pathological changes in testes, oxidative stress, autophagy	Wang et al., 2020 [135] Guan et al., 2021 [137]
	Goat sperm	10 μM for up to 12 h at 38.5 °C	60 μM for up to 12 h at 38.5 °C	Oxidative stress, sperm motility, survival rates, membrane integrity, mitochondrial activity, altered embryo development	Mao et al., 2018 [138]
	Mice	20–100 mg kg^−1^ b.w. day^−1^ for 1 week	1.2 mg kg^−1^ b.w. day^−1^ for 1 week	Memory impairment of the F1-F2 generation, brain activity, expression of GST and CAT, Cd uptake	Halder et al., 2019 [139]
Hesperetin	Rat	40 mg kg^−1^ b.w. by oral administration	3 mg kg^−1^ by s.c. injection for 21 days	Acetylcholinesterase, ROS, protein carbonylation, SOD, CAT and DPx, GSH, vitamin C, total sulfhydryl groups, apoptotic markers, mitochondrial dysfunction	Shagirtha et al., 2016 [142]
Anthocyanins	Rat	10 mg kg^−1^ b.w. by stomach tube for 30 days	4 μg kg^−1^ b.w. by stomach tube for 30 days	Cd accumulation in the liver and kidney	Kowalczyk et al., 2003 [145]
	Mice	500 mg kg^−1^ day^−1^ for up to 30 days	5 mg kg^−1^ day^−1^ for up to 30 days	Levels of gonadotropins, luteinizing hormone and follicle stimulating hormone, Gnrh1 gene expression	Li et al., 2019 [146]
Naringin	HepG2 cells	5 μM for 24 h	50 μM for 24 h	Alteration of antioxidant system, cytotoxicity, redox homeostasis, mitochondrial membrane potential, apoptosis, SOD, GST, CAT activities, lipid peroxidation	Rathi et al., 2017 [148]
	Human lymphocytes	1–2 mg mL^−1^ for 24 h	20 and 40 μM for 24 h	Chromosomal aberrations, ROS, antioxidant metabolism	Yılmaz et al., 2012 [149]
Curcumin	Mice	0.14 mM kg^−1^ b.w. by gavage for 3 days, or 100 mg kg^−1^ b.w. by single i.p. injection	33 μM kg^−1^ or 5 mg kg^−1^ b.w. by s.c. injection 1 or 24 h after curcumin treatment	Antioxidant enzymes activity, levels of total glutathione and thiol, MDA, H_2_O_2_,	Eybl et al., 2006 [151], Momeni et al., 2020 [152]
Carvacrol	PC12 cells	100 μM for 48 h	10 μM for 48 h	Growth retardation, glutathione and glutathione reductase, caspase 3, cytochrome c, apoptosis inducing factor, mTOR, protein kinase B, nuclear factor kappa-light-chain-enhancer of activated B cells, extracellular signal-regulated kinase-1, nuclear factor erythroid 2-related factor 2	Banik et al., 2019 [154]
Ferulic acid	Rat	50 mg kg^−1^ b.w. orally administered for 15 or 30 days	10 mg kg^−1^ b.w. for 15 and 30 days by s.c. injection	Body weight and serum total protein contents, histopathological damage, AST, ALT, ALP and LDH, uric acid, urea, urea nitrogen, and creatinine content), lipid hydroperoxides, MDA, protein carbonyl content, total oxidant status, and oxidative stress index, total thiols, total antioxidant concentration, SOD, CAT, and GPx, GSH and total free sulfhydryl groups, TNF-α, COX-2, and HSP70 proteins	Sanjeev et al., 2019 [155]
Vanillic acid	*Oryza sativa* L.	50 μM seedlings exposure for 72 h	1 and 2 mM seedlings exposure for 72 h	Photosynthetic pigment, osmotic status, biomass accumulation, growth, ROS, ascorbate pool size, antioxidant, and glyoxalase systems, phytochelatin content	Bhuyan et al., 2020 [159]
Salicylic acid	*Solanum tuberosum* L.	600 μM foliar spray exposure for 10 days	200 μM foliar spray exposure for 10 days	RWC, antioxidant enzymatic mechanism pathway, chlorophyll, proline content, MDA, H_2_O_2_, and superoxide anion radicals	Li et al., 2019 [161]
Abscisic acid	*Lactuca sativa* L.	10 μg L^−1^ sprayed on leaves	100 μM in hydroponic nutrient solution	Oxidative stress, H_2_O_2_, MDA, photosynthesis, mineral nutrients content, plant biomass	Dawuda et al., 2020 [163]
Melatonin	*Brassica napus* L. *Malva parviflora* L.	15, 50 or 100 µM in the nutrition solution	25 µM for 5 days and 50 µM for 8 days	Photosynthesis, SOD, CAT, peroxidase, ascorbate peroxidase, proline, chlorophyll and anthocyanin content, MDA, H_2_O_2_	Sami et al., 2020 [165], Tousi et al., 2020 [166]
	*Malus domestica* L.	100 µM for up to 20 days	30 µM for up to 20 days	Cd translocation, antioxidant enzymes activity, root Cd uptake, and leaf Cd accumulation	He et al., 2020 [167]
	*Brassica rapa* subs. *pekinensis*	100 µM sprayed of leaves once a day	20 µM in the nutrient solution for 8 days	Nitric oxide, expression of the transporter gene IRT1, Cd absorption	Ni et al., 2018 [168]
	*Catharanthus roseus* (L.) G. Don	100 μM foliar spray	50, 100, 200 mg kg^−1^ soil for 30 days	Phytoremediation efficiency, shoot biomass and chlorophyll content, POD and CAT activities, electrolyte leakage	Nabaei and Amooaghaie 2020 [170]
	*Solanum lycopersicum* L.	100 μM foliar spray for 15 days every 5th day	100 μM for 15 days	Sulfur metabolism, caffeic acid O-methyltransferase (COMT) gene	Hasan et al., 2019 [171]
	Mushrooms	50, 100, or 200 μM in the growth medium for 5 days	2, 5, and 8 μM in the growth medium for 5 days	Amino acid and glutathione metabolism, oxidation-reduction processes, metal, and ROS	Gao et al., 2020 [172]
	Mice	25 mg kg^−1^ for 14 days by i.p. injection	5 mg kg^−1^ for 14 days by i.p. injection	Ovulation dysfunction and ovarian injury, pathohistological damage	Yang et al., 2019 [173]
	Mice	10 mg kg^−1^ by i.p. injection for 3 days before Cd administration	2 mg kg^−1^ by single i.p. injection	Hepatocellular damage, ALT/AST enzymes, antioxidant activity, thioredoxin-interacting protein, TXNIP-NLRP3 inflammasome pathway	Cao et al., 2017 [177]
	Ovarian cancer cells	1 μM for 48 h in growth medium	1–100 nM for 48 h in growth medium	Estradiol (E2)-derived proliferation	Ataei et al., 2018 [174]
	Rat	3 mg L^−1^ in drinking water for 1 month	50 mg L^−1^ in drinking water for 1 month	Mineral and organic components, Ca^2+^ level, bone damage and histological alterations	Knani et al., 2019 [175]
	Rat	4 mg kg^−1^ 30 min prior to Cd administration	1 mg kg^−1^ by i.p. injection for 8 weeks	Memory and learning disabilities, NO and lipid peroxidation, CAT and SOD, Cd-induced neuronal loss	Lamtai et al., 2021 [176]
	Human adipose cells	10 nmol L^−1^–50 μmol L^−1^ in growth medium for 4 to 72 h	0.25 to 10 μmol L^−1^ in growth medium for 4 to 72 h	Osteogenic differentiation properties, adipogenic differentiation	Knani et al., 2019 [175]
Fulvic acid	*Lactuca sativa* L.	0.5 g L^−1^ in hydroponics for 2 weeks	20 μM in hydroponics for 2 weeks	Nutrient elemental imbalance, pigment content, photosynthesis, photosystem PSII, ROS, antioxidant capacity	Wang et al., 2019 [181]

## Data Availability

Not applicable.
